# Mental Health Status of Psychogeriatric Patients During the 2019 New Coronavirus Disease (COVID-19) Pandemic and Effects on Caregiver Burden

**DOI:** 10.3389/fpsyt.2020.578672

**Published:** 2020-11-17

**Authors:** Camila T. Penteado, Julia C. Loureiro, Marcos V. Pais, Cláudia L. Carvalho, Lívea F. G. Sant'Ana, Leandro C. L. Valiengo, Florindo Stella, Orestes V. Forlenza

**Affiliations:** ^1^Laboratory of Neuroscience (LIM-27), Departamento e Instituto de Psiquiatria HCFMUSP, Faculdade de Medicina da Universidade de São Paulo, São Paulo, Brazil; ^2^Instituto Nacional de Biomarcadores em Neuropsiquiatria (INBioN), Conselho Nacional de Desenvolvimento Científico e Tecnológico, São Paulo, Brazil

**Keywords:** coronavirus disease 2019 (COVID-19), pandemic, psychogeriatrics, Down syndrome, mental health, caregiver distress, caregiver burden

## Abstract

**Introduction:** There is a growing awareness about the noxious effects of the 2019 Coronavirus Disease (COVID-19) pandemic on the mental health of the elderly. However, there is limited information from clinically driven research. The objectives of the present study were to examine the magnitude of psychiatric symptoms and to determine their association with caregiver distress, in a cross-section of community-dwelling older adults and a subsample of aging adults with Down syndrome (DS) attending a psychogeriatric service in São Paulo, Brazil.

**Method:** Telephone-based interviews and electronically filled self-assessment questionnaires were used to collect information from patients and caregivers, addressing their impressions and concerns about the pandemic and related effects on the patient's emotional state and behavior. Clinical information was obtained from hospital charts, medical records, and psychometric tests administered through telephone interviews [Hospital Anxiety and Depression Scale (HADS) and Neuropsychiatric Inventory Questionnaire (NPI-Q)].

**Results:** We included 100 consecutive participants, comprising 71 older adults with psychogeriatric/neurocognitive disorders and 29 aging adults with DS. Higher HADS and NPI-Q scores were significantly associated with caregiver distress (*p* < 0.05) in both groups. Correlation analyses indicated strong, positive associations between caregiver burden and scores in HADS anxiety (HADS-A) and HADS depression (HADS-D) scales in the subsamples of euploid and DS subjects. Higher NPI-Q scores in the former group were also correlated with caregiver distress, with stronger associations for neuropsychiatric symptoms. Similar findings were observed among DS subjects. ANOVA tests indicated significant associations between NPI-Q scores and caregiver distress among dementia patients, as well as with HADS scores. Similar results were found after multiple linear regressions; as such, among the elderly subsample, higher scores in HADS-A (*p* = 0.002) and HADS-D (*p* = 0.001) predict a significant impact on caregiver burden (*p* < 0.00001, *R*^2^ 0.46); taking into consideration caregiver burden as a dependent variable and NPI-Q total score as an independent variable, we obtained significant strong prediction values for either DS (*p* < 0.00001, *R*^2^ 0.95) or elderly adults (*p* < 0.00001, *R*^2^ 0.88).

**Conclusion:** During the COVID-19 pandemic, patients with neurocognitive disorders present with clinically relevant neuropsychiatric symptoms, with significant impact on caregiver distress. Apathy, aberrant motor behavior, sleep disorders, and psychoses were the main psychopathological domains, which had determined caregiver burden worsening.

## Introduction

The 2019 new coronavirus disease (COVID-19) was declared a “Public Health Emergency of International Concern” (PHIC) on January 30, 2020, and the World Health Organization (WHO) declared the “COVID-19 pandemic” on March 11, 2020 (1). In Brazil, the first case of COVID-19 was reported in São Paulo, on February 26, 2020, by the Brazilian Ministry of Health. To date, cases in Brazil surpass 1.3 million with more than 58,000 confirmed deaths (2). Social and economic issues, combined with a heterogeneous (often fragile) and overloaded healthcare system, have been major concerns over the past 4 months. The potentially catastrophic impact of the pandemic, combined with limitations derived from the disease containment measures, imposed significant mental health challenges to the population. Widespread concerns often arise in the pandemic crises, such as the persistent determination for self-protection, changes in daily routine, abrupt interruption of activities outside the home, and use of masks that make it difficult to recognize people's faces. In addition, problems of social interactions and emotionally charged reactions tend to erupt with the increase in the number of people living together in the same space. Together, these events may predispose to the exacerbation of psychiatric symptoms in vulnerable patients.

Recent studies have demonstrated that the elderly represent one of the most vulnerable populations to present with behavioral and mental disorders as a consequence of the COVID-19 pandemic (3), along with other at-risk subgroups such as the homeless (4), immigrant workers (5), pregnant women (6), and people with preexisting mental illnesses (7, 8). Although social isolation and interpersonal distancing have been adopted worldwide as measures to stall the dissemination of the viral infection, these measures also may exert a negative impact on the mental health of the population, particularly among the elderly. In addition to representing a sudden and significant change in their daily routine, social isolation may trigger feelings of abandonment and loneliness, therefore increasing the risk of depression (9). Older adults who live alone and are nonetheless still autonomous may become dependent on the help from relatives and neighbors for the provision of basic supplies, with subsequent impact on their mental health. In contrast, an increase in caregiver emotional burden is expected as a consequence of social isolation and other restrictions imposed by the pandemic on the mental health of less autonomous persons.

The COVID-19 pandemic has also been associated with an increase in the incidence of mental disorders (10), possibly occurring at a higher rate in severe forms of the disease (11). Psychological distress and psychiatric manifestations range from mild symptoms, such as feelings of frustration, distress, fear, and anger, to moderate and severe symptoms of anxiety, depression, sleep disorders, and worsening of preexisting psychiatric disorders (12, 13). In addition, the media overexposure of ambiguous and incomplete information regarding the pandemic, including untruthful and alarmist data, tends to generate feelings of uncertainty and to aggravate behavioral and mental disorders (11).

Studies with community-dwelling older persons reported rates of depression and anxiety symptoms up to 37.1% (12, 14), again occurring especially in those severely affected by the COVID-19 (11). Psychic suffering related to fear of dying from COVID-19 (4), as well as worsening of preexisting psychiatric disorders as consequence of the restrictions imposed by the pandemic, impacts directly on vulnerable populations (15, 16). A population-based study using self-reported questionnaires showed that of 52,730 respondents, 35% reported the presence of pandemic-related psychic distress, especially occurring in adults aged between 18 and 35 and in the elderly (17). However, it is relevant to determine the distinct characteristics between mental disorders presented in clinical and community settings, between rural and urban populations.

People with Down syndrome (DS) are considered to be at an even higher risk of contamination by severe acute respiratory syndrome coronavirus 2 (SARS-CoV-2). The risk of contamination occurs due to the dysregulation of the autoimmune system caused by trisomy 21 since childhood and the presence of preexisting clinical comorbidities, such as diabetes, obesity, respiratory diseases, and heart disease. Despite the scarcity of scientific evidence of the impact of COVID-19 on DS, recent literature on this topic points to the existence of exacerbated immune dysfunction, increasing the rates of inflammatory cytokines and chemokines in people with DS compared to individuals with normal karyotype, therefore requiring additional monitoring and specialized care (18) during the pandemic. Genetic changes that occur on chromosome 21 in people with DS are also significant risk factors for cognitive decline and early-onset Alzheimer's disease (AD) (19). As the life expectancy of individuals with DS goes on increasing because of improved health care, education level, and social support, the risk of progressing to dementia, mainly due to AD, may also reach higher proportions (20). Therefore, we have decided to better understand if these people exhibit special behavioral features suggesting a distinct psychopathological pattern, possibly concomitant with cognitive decline, or perhaps if they reveal sufficient resilience to protect themselves with respect to mental health in the context of the COVID-19 pandemic. These changes resulting from premature aging expose them to an even greater vulnerability in the face of natural disasters and global crises such as the COVID-19 pandemic (21).

There is little doubt about the need for an appropriate provision of care for certain population subgroups at increased risk of experiencing psychological and psychiatric distress related to the pandemic. On the human resources end, teams of mental health professionals are being allocated to deal with the specific needs that emerge in certain subgroups. Training programs for community-based healthcare professionals and development of online assessment using different types of media have been largely recommended. Online surveys have been used in various settings to assess the effects of the pandemic on mental health; educational materials and self-help content, available free of charge on institutional websites and other communication environments, were developed with a focus on mental health. Finally, the provision of remote assistance and guidance (telemedicine) increased the reach of these emergency strategies (22).

A small number of investigations, most of them cross-sectional, about the impact of COVID-19 on mental health faced an unexpected limitation. In a prospective and longitudinal study, Wang et al. (23) applied a questionnaire through an online survey platform to 1,738 people from the general population in China, aged 12–59 years. The questionnaire was completed twice, with a 4-week interval between both assessments. The first evaluation detected moderate-to-severe stress in 8.1% of people, depression in 16.5%, and anxiety in 28.8%, interestingly with no significant changes compared to the second assessment despite pandemic sharply increasing in this period. Several protective factors contributed to mental health stability, including high level of confidence in doctors, perceived survival likelihood and low risk of contracting COVID-19, satisfaction with health information, and personal precautionary measures.

The present study is part of an ongoing clinical effort to provide medical care and psychological support to elderly outpatients at a university-based, tertiary hospital in São Paulo, Brazil (HCFMUSP), during the COVID-19 pandemic fight. This cohort comprises older adults with preexisting psychiatric and neurocognitive disorders (mostly AD) and a subsample of aging adults with DS. The search strategy used in this study was based on telephone interviews and online survey questionnaires, intended to characterize the emerging needs of our patients in view of the restrictions precluding regular hospital consultations. Our specific goals were (a) to determine the presence and severity of psychopathological symptoms in a community-dwelling group of older adults with neuropsychiatric conditions, for example, neurocognitive disorder, mood disorder, anxiety, psychotic symptoms, apathy, and sleep disorders, as well as behavioral disturbances of adult patients with DS; and (b) to determine the association between the aforementioned symptoms on caregiver burden, particularly in the presence of severe psychological and behavioral disturbances.

## Methods

### Study Group

The present study was designed with the general purpose of understanding pandemic-related mental changes in an at-risk population consisting of elderly patients with preexisting neuropsychiatric disorders and aging adults with DS. We decided to enroll these two groups in this COVID-19 study because (i) these two patient groups are indeed available in our psychogeriatric service and (ii) we understand that any clinical insights about the DS subgroup would be relevant, albeit distinct from euploid elders. The groups analyzed in this cross-sectional and exploratory study comprised a consecutive sample of patients routinely followed up by our Psychogeriatrics Team at the Instituto de Psiquiatria HCFMUSP, Brazil. Eligible subjects came from a relatively large community cohort, originally estimated at 500 individuals participating in this investigation of pandemic-related psychiatric disorders. Therefore, the results described in this article represent an interim analysis of a subsample of participants enrolled to date. At this moment, patients with dementia were considered as a single group, with no distinction between diagnostic types. All diagnoses disclosed in this study were based on DSM-V criteria, which were captured from the medical records.

### Ethics

This study was carried out in accordance with the Institute of Psychiatry HCFMUSP guidelines on clinical research practices, as well as the Helsinki Declaration. Prior to admission in the study, the patient provided written informed consent if he/her was cognitively preserved, or this agreement was authorized by his/her legal representative. It was also reviewed and approved by the Research Ethics Committee (Comitê de Ética em Pesquisa—CEP) of HCFMUSP under approval number 4.190.468.

### Assessment

We assembled via videoconferences a group of geriatric psychiatrists to build a questionnaire aiming to screen for (and monitor) the occurrence of psychiatric symptoms and changes in mental state of elderly patients with preexisting neuropsychiatric conditions during the first months of the Brazilian COVID-19 pandemic. This assessment instrument was designed to cover a wide range of mental health issues, including behavioral disturbances and emotional difficulties related to social isolation and daily routine acceptance; in addition, we assessed the caregivers' burden in light of the potential worsening of patients' neuropsychiatric symptoms. The full version of the study questionnaire is available to the readers as a Supplementary Material. We established that the informant had to be a close relative to the patient or a professional caregiver who maintained daily contact with him/her; therefore, enrollment was conditioned on the availability of an able informant.

#### Informant's Questionnaire

The assessment of changes in mental health status as a consequence of incident symptoms or relapse/worsening of preexisting conditions was captured by (i) specific questions dedicated to this aspect in the Informant's Questionnaire (online assessment) and (ii) by the perception of change in neuropsychiatric symptoms according to the clinician's judgment, captured by telephone interviews with the informants. In the first case, we used multiple-choice questions to address whether the informant had observed any recent changes in mental state or behavior that might be related to the COVID-19 crisis or if there had been any observable changes (from worsening to improvement) in the mental health state of the patient in the previous month. After these two introductory questions, we presented four additional multiple-choice questions to objectively address changes in sleep pattern, appetite, eating behavior, and weight. These questions were followed by a sequence of six check-box questions where the participant would be asked to indicate perceived changes in one or more symptoms, according to psychopathological domains (mood/affective, psychotic, behavioral/psychomotor, and cognitive). After each of these questions, the participant was able to express in writing his/her comments details about the aforementioned symptoms. As the majority of individuals in the study group had neurocognitive disorders, a set of questions was dedicated to estimate the informant's perception of changes in global cognition, functionality, and behavior.

#### Clinician's Questionnaire

Another questionnaire was prepared to assess distinct clinical aspects related to the patient's mental state in the context of the pandemic, for example, worsening of mood-related and behavioral symptoms, particularly if requiring the adjustment of the prescription of psychotropic drugs and medicines prescribed for the treatment of general medical conditions. We also assessed the difficulties faced by the family members or caregivers to manage behavioral disturbances presented by the patients during social isolation. A qualified member of our psychogeriatric team recovered data from the hospital charts, addressing the patients' medical records to complete this part of the questionnaire. Further, the Hospital Anxiety and Depression Scale (HADS) and Neuropsychiatric Inventory Questionnaire (NPI-Q) scale were administered by telephone interviews with the informants.

#### The HADS

The HADS (24) is an instrument widely used in different populations to detect the overall state and severity of anxiety and depression symptoms (25). The scale consists of two integrated subscales containing 14 mixed questions (seven for anxiety, HADS-A, and seven for depression, HADS-D). The items are focused on psychological manifestations in the last 2 weeks, excluding somatic signs and symptoms or avoiding dependence on physical diseases, such as fatigue, pain, headache, or dizziness (24, 26). Thus, this tool has been criticized for its overreliance on psychological domains as being the core symptoms of depression or anxiety (26). However, relevant clinical domains, for example, sleep disturbances, appetite disorders, and strange or delusional thoughts not inserted in the HADS, are apprehended through the NPI-Q, mentioned below.

The HADS was validated in a cohort of Brazilian people with a cutoff point of 8/9 for anxiety or depression (27) with specificity values of 93.7 and 72.6% for anxiety and 84.6 and 90.3% for depression, respectively (27). The scale has good internal consistency and case-finding properties (28).

#### The NPI-Q

The NPI-Q (29) has been administered to capture psychological and behavioral symptoms in patients with neurocognitive disorder and DS over the past month. This scale assesses 12 psychopathological domains commonly exhibited by patients with cognitive deterioration, focusing on severity, but not on the frequency of symptoms. The informant rates each psychopathological item according to the severity of the patient's symptoms as well as to himself suffering from emotional distress. Instructions are provided to guide the respondent to complete the questionnaire and anchor points for ratings. Thus, each item requires an answer like “yes” or “no.” Subsequently, the severity of this item is classified as “mild” (1), “moderate” (2), or “severe” (3). The total NPI-Q severity ranges from 0 to 36. In addition, the NPI-Q assesses the primary caregiver distress related to each patient's response. The caregiver's distress varies from “not emotionally stressful” (0) to “extremely stressful” (5), with a total score extending from 0 to 60.

The NPI-Q has good test–retest reliability and convergent validity (30). The Brazilian version of the NPI-Q depicted a reliability of 0.97 for the severity subscale and 0.92 for the distress subscale, with moderate internal consistency for the severity subscale and strong consistency for the distress subscale (31). Therefore, the structural properties of the NPI-Q allow a comprehensive measurement of neuropsychiatric symptoms of patients with dementia in clinical practice or research settings (31).

### Statistical Analysis

Descriptive statistics were calculated for sociodemographic characteristics, access to private or public health assistance, level of worry about the pandemic, concern-related variables, knowledge and level of compliance to precautionary measures, impact on preexisting mental disorders, complaints of fear and loneliness, presence of a caregiver or family member at home, and physical and psychological symptoms reported. Group comparisons of demographic variables used ANOVAs for continuous measures and Fisher's exact test. Spearman correlations were used to calculate the associations between sociodemographic characteristics, physical symptoms, symptoms of anxiety and depression, and caregiver burden. We used multiple linear regressions as NPI-Q subitems as the dependent variable and gender, HADS-A and HADS-D scores, and age as the independent variables. The same was made for the caregiver burden as the dependent variable and age, gender, HADS-A and HADS-D scores, and all NPI-Q total scores and subitems as the independent variables. All analyses were performed for each group separately (DS subjects and elderly adults). The significance level considered was *p* < 0.05, and all statistical analyses were performed using Stata version 15.0 for Mac.

## Results

Our sample consisted of 71 elderly adults and 29 DS patients. As displayed in Table 1, euploid subjects were considerably older than trisomic (DS) subjects and predominantly married; most of them had access to private health insurance assistance and were, for the most part, retired from previous occupations. As for the DS cohort, participants were evenly distributed between genders; they were all single, mostly dependent on the public health system; and the vast majority had never worked before, fully relying on family support.

**Table 1:** Socio-demographic characteristics of both cohorts, elderly adults and DS patients.

**Socio-demographic features**	**Elderly *n* = 71 (SD)**	**DS *n* = 29 (SD)**
Age (years)	76.8 (8.7)	43.3 (13.4)
Gender M/F	22/ 49	14/ 15
Education (years)	12.8 (7.4)	13.4 (12.0)^*^
**Marital status**		
Married	53.0%	-
Separated/Widowed	38.8%	-
Single	8.2%	100%
**Health assistance**		
Public health system only	20.4%	73.7%
Private health insurance	79.6%	26.3%
**Occupation**		
Retired	75.5%	5.0%
Working	12.2%	10.0%
Unemployed	-	5.0%
Never worked	12.2%	80.0%

DS, down syndrome; mean values for age and education; SD, standard deviation.

* *Down syndrome patients' years of education refer to special schooling*.

Preexisting neuropsychiatric conditions were identified and are described in Table 2. The main diagnoses consisted of mild neurocognitive disorder [i.e., mild cognitive impairment (MCI) with either amnestic or multiple domain characteristics], major neurocognitive disorder (i.e., AD, vascular dementia, mixed dementia, frontotemporal dementia, and semantic dementia), and affective disorder (i.e., bipolar disorder and depression with or without related apathy syndrome).

**Table 2:** Clinical characteristics of both sub-samples, elderly adults and DS patients, as observed previously to the pandemic outbreak.

**Clinical features previous to the pandemic**	**Elderly (*n* = 71)**	**DS (*n* = 29)**
Presence of NCD	90.1% (64)	34.4% (10)
Severity of NCD		
MCI	40,6% (26)	30.0% (3)
Mild/Moderate dementia	35.9% (23)	60.0% (6)
Severe dementia	25.0% (16)	10.0% (1)
Depression	50.7% (36)	3.4% (1)
Bipolar affective disorder	2.8% (2)	0% (0)
Presence of CVD	78.9% (56)	55.2% (16)

*DS, down syndrome; NCD, neurocognitive disorder; MCI, mild cognitive impairment; CVD, cardiovascular disease*.

**Figure 1:**
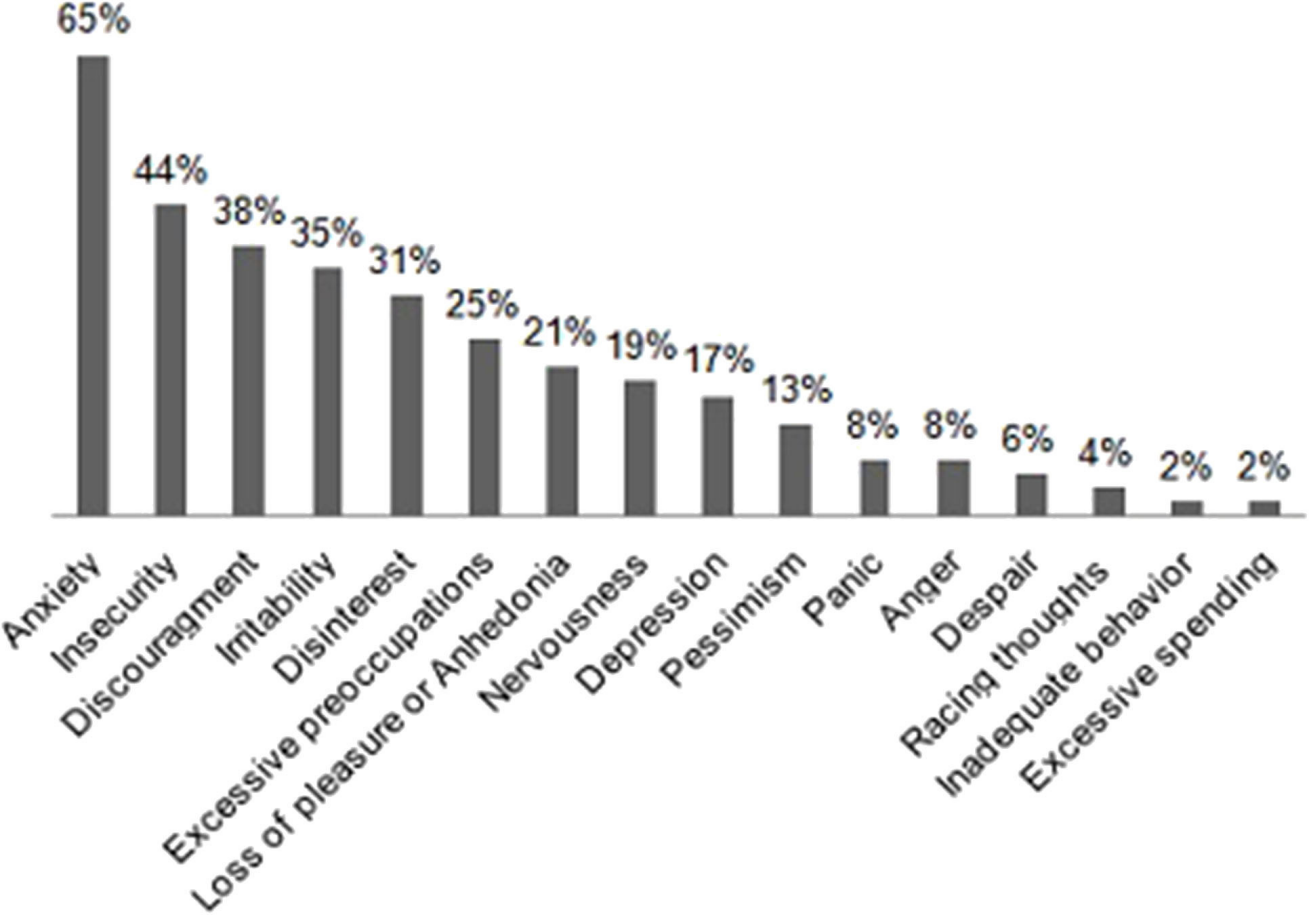
Prevalences of psychiatric manifestations occurring during the COVID-19 crisisas observed in the elderly group (*n* = 48).

**Figure 2:**
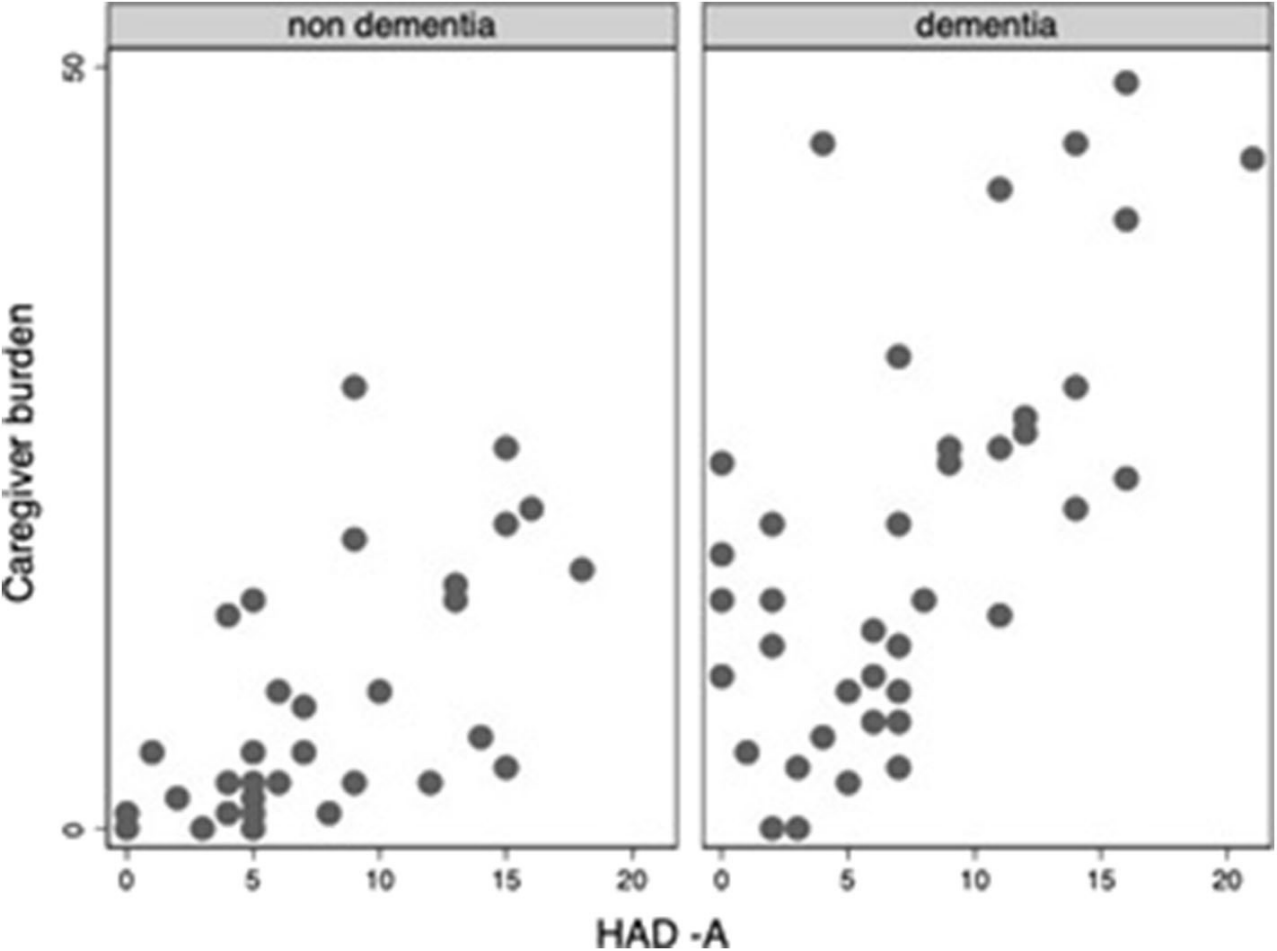
Scatter plot of Caregiver burden and HAD-A scorcs among elderly adults (with and without dementia).

**Figure 3:**
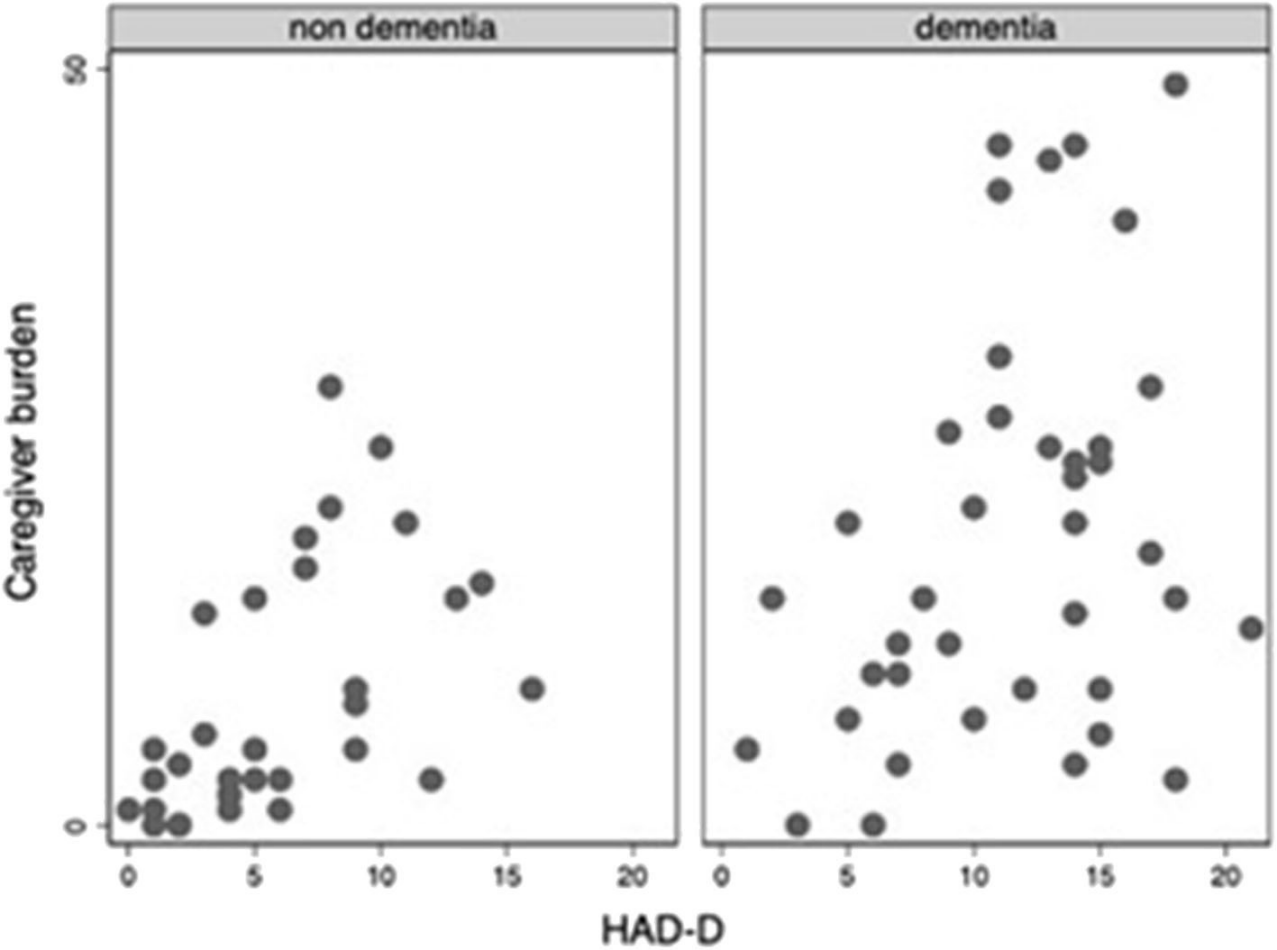
Scatter plot of Caregiver burden and HAD-D scorcs among elderly adults (with and without dementia).

**Figure 4:**
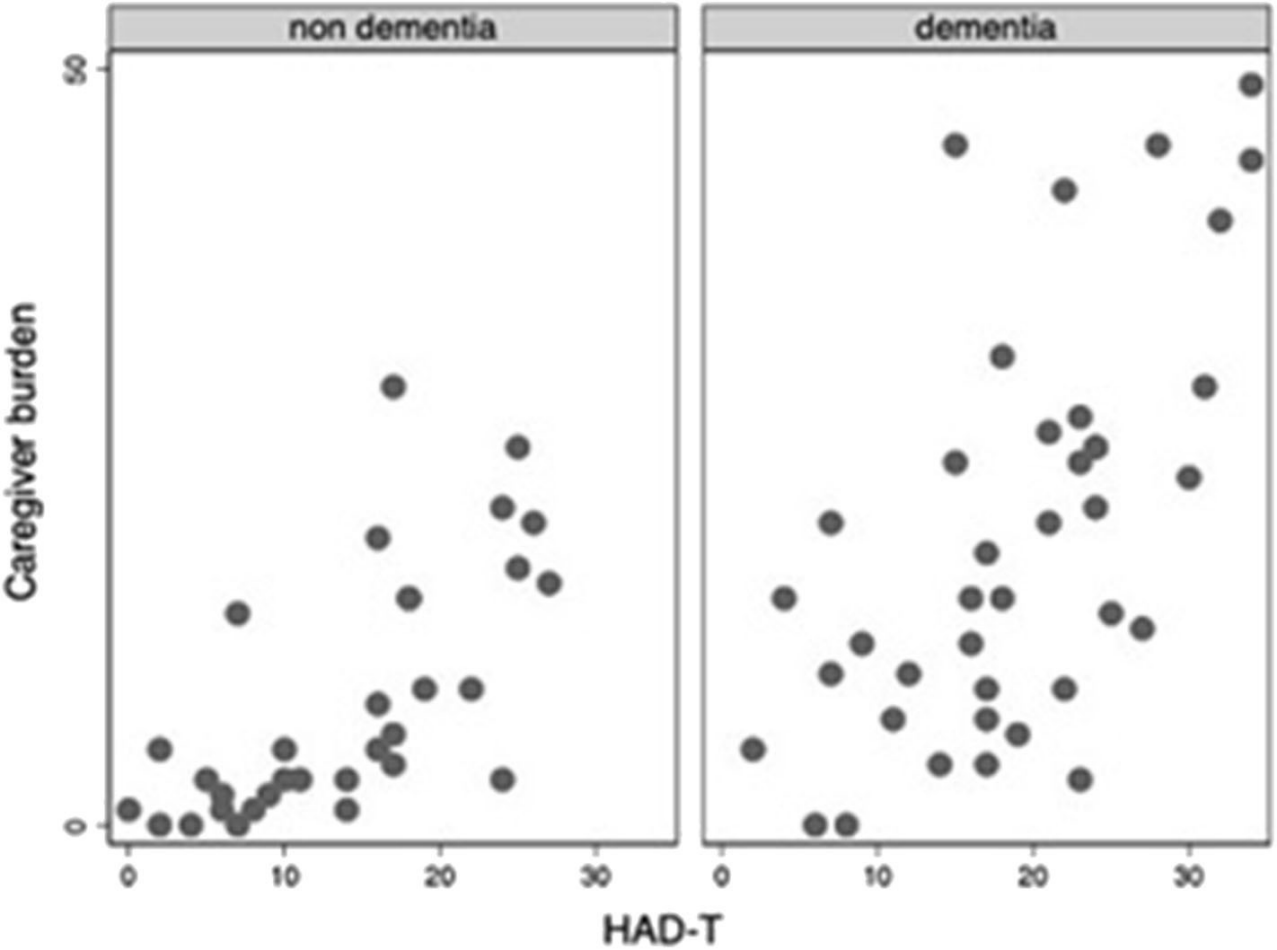
Scatter plot of Caregiver burden and HAD-T scorcs among elderly adults (with and without dementia).

Throughout the COVID-19 pandemic outbreak, we were able to recognize emerging or aggravating neuropsychiatric features such as mood symptoms, sleep problems, and psychotic disturbances. The most prevalent self-referred psychiatric symptoms in the elderly group were anxiety (65%), feeling of insecurity (44%), discouragement (38%), and irritability (35%) (Figure 1). Mood symptoms with depressive and anxious traits were measured by means of the HADS, yielding a maximum of 21 points for each subscale (HADS-A and HADS-D) and a total of 42 points for total HADS score (HADS-T). Mean scores for elderly adults and DS patients were, respectively, 7.6 (SD: 5.1) and 3.9 (SD: 3.5) for HADS-A; 8.9 (SD: 5.3) and 4.8 (SD: 5.1) for HADS-D; and 16.5 (SD: 8.4) and 8.8 (SD: 7.9) for HAD-T. Figures 2–4 display scatter plots that illustrate how higher scores in the HADS-T and its subscales correlate with increased caregiver burden as well as with the severity of the neurocognitive diagnosis. Patients with dementia, therefore, present with worse HADS scores and higher caregiver impact.

**Figure 5:**
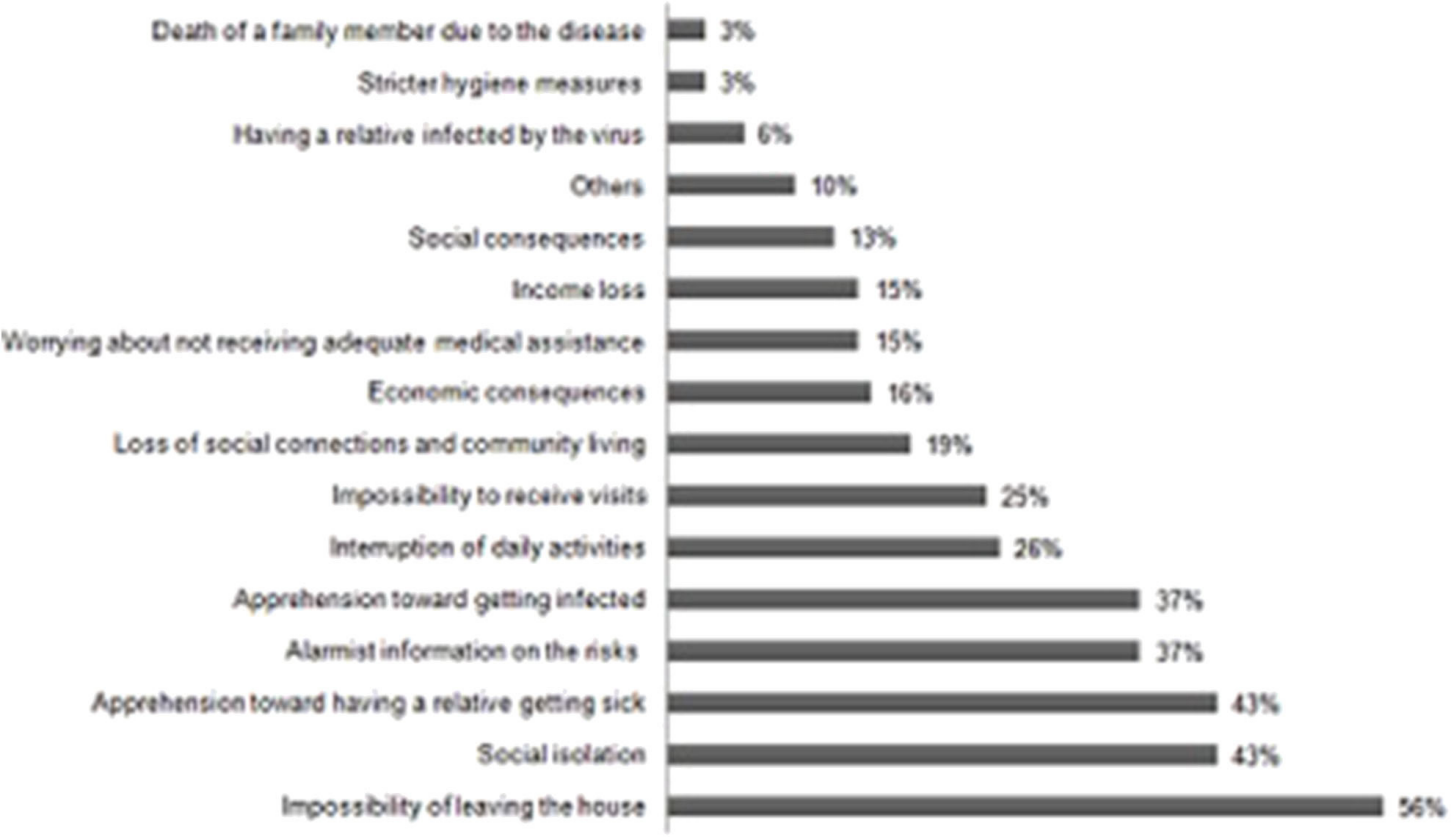
Specific aspects of the COVID-19 pndemic that interfere with mental health (*n* = 68).

With respect to specific and inherent aspects of the COVID-19 pandemic that might have contributed to the emergence or aggravation of mental health disturbances, elderly participants indicated the “impossibility of leaving the house” as the one responsible for greater impact (56%). It was closely followed by “social isolation” (43%); “apprehension toward the possibility of a relative getting sick” (43%); “alarmist information on the exposure risks” (37%); and “apprehension oneself might be infected” (37%) (Figure 5).

**Figure 6:**
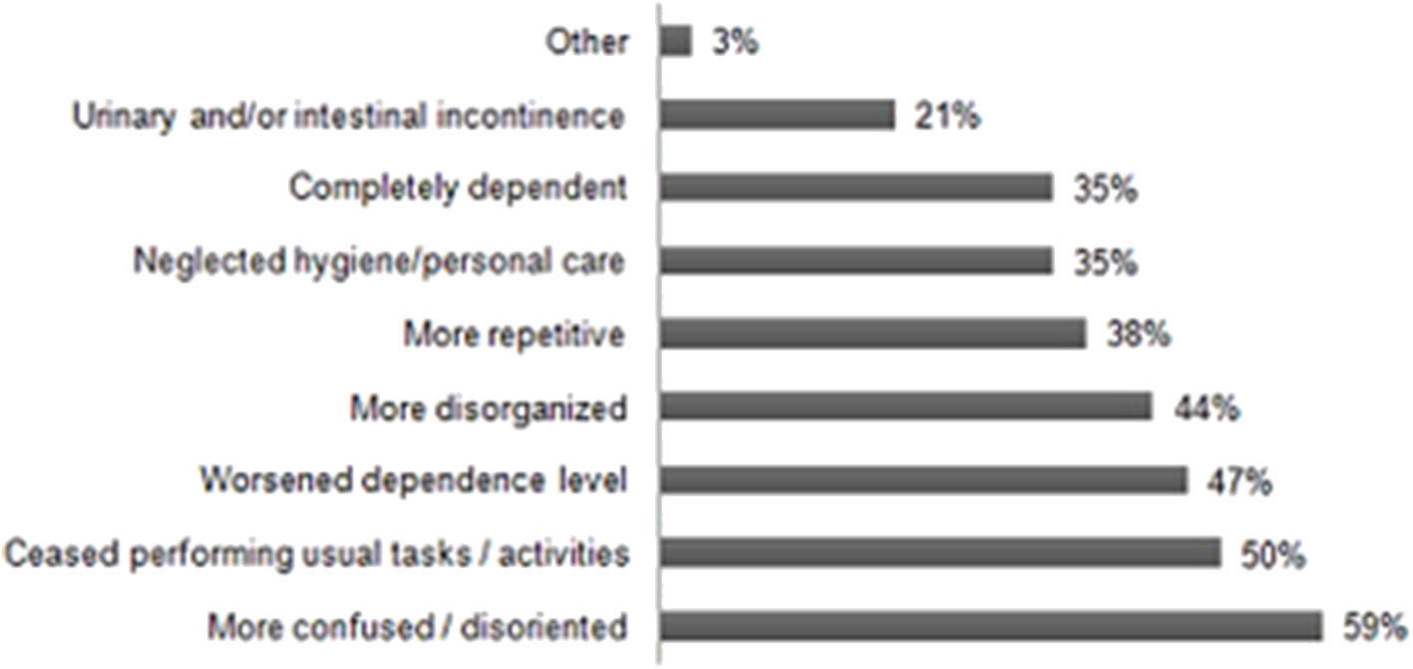
Changes in cognitive-functional performance of elderly patients occuring during the COVID-19 pandemic (n = 34).

According to caregivers' reports, changes in cognitive status were observed in 34 elders, suggesting cognitive decline. Among the most prevalent new or evolutive symptoms, “worsened disorientation and confusion” accounted for the higher prevalence, occurring in 59% of patients, followed by “ceased performing usual tasks or activities” (50%), “higher dependence” (47%), “worsened disorganization” (44%), and being “more repetitive” (38%), which were also frequently reported (Figure 6).

ANOVA tests indicated statistically significant differences in mean HADS and NPI-Q scores and subscores when comparing the subsamples of elderly subjects with and without dementia (HADS-A, *p* = 0.001; HADS-D, *p* = 0.002; HADS-T, *p* = 0.01; NPI-Q-psychosis, *p* < 0.0001; NPI-Q-apathy, *p* = 0.004; NPI-Q-aggressiveness, *p* < 0.0001; NPI-Q-movement disorders, *p* < 0.0001; NPI-Q-sleep, *p* < 0.0001).

Spearman's correlation tests demonstrated statistically significant correlations between psychometric test scores (HADS and NPI-Q) and caregiver burden. In the subsamples of euploid elders, these correlations were strong (HADS-T, 0.87; HADS-A, 0.78; and HADS-D, 0.80) and statistically significant (*p* < 0.05), whereas in the subsample of DS, these correlations were moderate to strong (HADS-T, 0.66; HADS-A, 0.52; and HADS-D, 0.55, *p* < 0.05 for all tests). Similar correlations were observed between the NPI-Q subscores (psychosis, aggressiveness, depression, apathy, irritability, aberrant movement disorder, and sleep disturbances) and the degree of caregiver burden. Among euploid elders, strong correlations were found for apathy (0.81), aberrant movement disorders (0.78), sleep disturbances (0.71), and psychosis (0.69); moderate correlations were found for irritability (0.66), depression (0.63), and aggressiveness (0.59). Among DS participants, correlations were strong for aberrant movement disorders (0.69), sleep disturbances (0.65), and irritability (0.67); and moderate correlations were found for aggressiveness (0.62), depression (0.52), and psychosis (0.57).

Multiple linear regressions demonstrated significant associations between NPI-Q scores and HADS-A and HADS-D scores in both groups. Tables 3, 4 display the results of this complete regression analysis for each subsample (DS and elderly adults). Also, among the elderly subsample, higher scores in HADS-A (*p* = 0.002) and HADS-D (*p* = 0.001) predict a significant impact on caregiver burden (*p* < 0.00001, *R*^2^ 0.46). Likewise, the regressions between caregiver burden as the dependent variable and NPI-Q total score as the independent variable showed significant strong prediction values for either DS (*p* < 0.00001, *R*^2^ 0.95) or elderly adults (*p* < 0.00001, *R*^2^ 0.88). Table 5 displays the complete regression analysis among caregiver burden and each NPI-Q domain for both subsamples.

**Table 3:** Multiple linear regressions: NPI-Q domains × age, gender, HAD-A score and HAD-D score of the DS subsample.

**NPI-Q Domains**	***F***	***P*-value**	***R*^2^**	**pAge**	**pGender**	**pHAD-A**	**pHAD-D**
Psychosis	11.71	**0.0002**	0.7698	0.328	0.391	0.343	**0.007**
Aggressiveness	1.93	0.1617	0.3551	0.844	0.757	0.876	0.079
Depression	7.69	**0.0017**	0.6873	0.334	0.452	0.513	**0.004**
Apathy	6.17	**0.0045**	0.6379	0.584	0.831	0.113	0.059
Irritability	4.21	**0.0192**	0.5460	0.316	0.544	0.724	**0.008**
AMD	4.73	**0.0126**	0.5748	0.556	**0.024**	0.056	0.588
Sleep dist.	1.9	0.1662	0.3520	0.700	0.122	0.090	0.711
Apetite dist.	1.59	0.2323	0.3120	0.285	0.314	0.129	0.379

*DS, down syndrome; AMD, aberrant movement disorders; dist., Disturbances*.

*The bold values refer to those with statistical significance*.

**Table 4:** Multiple linear regressions: NPI-Q domains × age, gender, HAD-A scores and HAD-D scores of the elderly adults subsample.

**NPI-Q domains**	***F***	***P*-value**	***R*^2^**	**pAge**	**pGender**	**pHAD-A**	**pHAD-D**
Psychosis	3.78	**0.0104**	0.2696	0.070	0.552	0.175	**0.033**
Aggressiveness	4.42	**0.0046**	0.3012	**0.047**	0.812	**0.004**	0.230
Depression	2.17	0.0892	0.1748	0.754	0.515	0.326	**0.038**
Apathy	6.61	**0.0003**	0.3919	0.576	0.739	0.194	**>0.0001**
Irritability	8.13	**0.0001**	0.4423	0.396	0.814	**>0.0001**	0.097
AMD	4.54	**0.0040**	0.3068	0.111	**0.043**	0.089	**0.043**
Sleep dist.	1.69	0.1697	0.1419	0.957	0.308	0.376	0.061
Apetite dist.	0.44	0.7782	0.0413	0.238	0.902	0.970	0.492

*AMD, aberrant movement disorders; dist., disturbances*.

*The bold values refer to those with statistical significance*.

**Table 5:** Multiple linear regressions: caregiver burden × NPI-Q domains scores of both groups.

**DS**	**Caregiver burden**	**Elderly adults**	**Caregiver burden**
***F***	540.42	*F*	32.93
***P*****-value**	**< 0.00001**	*P*-value	**<0.00001**
*R*^2^	0.99	*R*^2^	0.92
pPsychosis	**0.003**	pPsychosis	**0.000**
pAgressiveness	**0.000**	pAgressiveness	0.115
pDepression	**0.004**	pDepression	0.095
pApathy	**0.012**	pApathy	0.100
pIrritability	0.161	pIrritability	0.052
pAMD	0.335	pAMD	**0.002**
pSleep dist.	**0.000**	pSleep dist.	**0.036**
pAppetite dist.	0.307	pAppetite dist.	0.205

*DS, down syndrome; AMD, aberrant movement disorders; dist., disturbances*.

*The bold values refer to those with statistical significance*.

Pharmacological interventions to deal with emerging neuropsychiatric symptoms were required only in a minority of the cases, that is, 28.2% (*n* = 20) of euploid elders and 3.4% (*n* = 1) of DS subjects; whenever required, these procedures were largely due to sleep complaints (FET 0.016, *p* = 0.038).

As for adaptability, the elderly subjects were more prone to cope well with and willingly follow governmental recommendations when their neurocognitive diagnoses were less severe (0.52) and when their level of insight toward the pandemic was higher (0.52). Also, with reference to insight, elders with a better comprehension of the pandemic situation demonstrated higher levels of reactivity (0.63), meaning they coped better and reacted more favorably in compliance with official recommendations. Inverse significant correlations were found between the severity of caregiver impact and DS patients' level of insight on the pandemic (−0.50), the patient's concern about his/her own health (−0.52), and level of reactivity toward respecting hygiene measures (−0.61). Higher insight was strongly correlated with reactivity (0.92), suggesting these patients were more prone to be alert and follow sanitary recommendations.

## Discussion

The present study was designed to monitor the mental state of the clients of a psychogeriatric clinic in São Paulo, Brazil, during the COVID-19 pandemic, using remote assessment methods. Our *a priori* hypothesis was that the restrictive measures related to the pandemic crisis could lead to a worsening of neuropsychiatric and behavioral symptoms, particularly in vulnerable subgroups. We found that mood-related symptoms were the most frequent complaints, reported by 65% of respondents. Based on data provided by the NPI-Q, among old patients, apathy, aberrant motor behaviors, sleep disorders, and psychotic symptoms compose a psychopathological constellation of symptoms commonly causing relevant caregiver emotional distress and creating considerable challenges for daily caring in quarantine. Regarding the impact on mental health, these manifestations were followed by others, such as irritability and concern with adaptability to new psychosocial demands. Therefore, symptoms of anxiety and depression, respectively depicted by HADS-A and HADS-D, significantly correlated with caregiver routine burden. Interestingly, correlations between psychopathological manifestations and caregiver distress were higher in the dementia group according to ANOVA tests, meaningfully for HADS-A, for HADS-D, and for several NPI-Q domains, such as psychosis, apathy, aggressiveness, aberrant movement disorders, and sleep.

In this study, apathy was a crucial source of impact on caregiver distress in the COVID-19 outbreak. It is a pervasive symptom affecting mostly patients with cognitive decline and includes a reduction in goal-directed activities concerned with behavior, cognition, emotions, and social interaction (32). Given the data described in the present work, apathy was a common neuropsychiatric symptom among patients with dementia, surely as a preexisting manifestation at the beginning of the pandemic. Our findings are in agreement with results reported by Lara and colleagues (33), who documented apathy as one of the most common psychopathological domains among patients with MCI or AD during the COVID-19 outbreak. Even though apathy can be separated from major depression because of distinct psychopathological features and different neurobiological ways, a substantial proportion of individuals may share clinical symptoms from both conditions (32).

Agitation was another psychopathological domain affected among old patients in the current study. This phenomenon was observed across all cognitive stages of dementia and encompasses erratic or repetitive behaviors, wandering, chaotic attitudes, and threats of verbal or physical aggression, causing critical impact on the well-being of patients and caregivers. Frequently, agitation is closely related to psychotic symptoms like delusions and hallucinations and seems to be predictive of a more severe course of cognitive impairment (34). Agitation and psychotic symptoms may coexist, and such co-occurrence subsequently aggravates behavioral disturbances and dangerous consequences for both the patient and caregiver (35).

Sleep complaints are characterized by trouble in initiating sleep, nighttime behavior disturbances, and daily somnolence. These symptoms were relevant complaints in old patients from our sample, also generating emotional charged reactions in caregivers. Concerning this issue, Casagrande et al. (36) conducted an investigation in a large Italian population by a web-based cross-sectional survey, broadcasted through different platforms and mainstream social media during the pandemic. They reported poor sleep quality, besides other clinical symptoms such as higher levels of generalized anxiety and greater psychological distress related to the COVID-19 outbreak. In particular, they emphasized a consistent connection between sleep disorders and behavioral disturbances, emotional dysregulation, anxiety, and depressive symptoms. Sleep fragmentation and loss of efficiency were common. In addition, not only did patients have sleep problems in the pandemic, insomnia is also a major concern for the medical staff due to the constant threat of contamination, being present in more than one-third of the clinical care team (37). Although there is insufficient evidence of direct causality between insomnia and viral contamination, it has been documented that shorter sleep duration, confirmed by wrist actigraphy measurement, increases the reduction in the natural immune response and predicts risks for infectious illnesses (38).

Psychotic symptoms frequently emerge mainly in the later stages in neurodegenerative diseases and have been associated with agitation episodes and more rapid global deterioration, causing serious and dangerous repercussion in both patients and caregivers (34). Outcomes from our investigations confirmed that delusions and hallucinations of old patients actually generate relevant emotional distress for caregivers in the current COVID-19 outbreak.

The elderly group also revealed important insecurity feelings and was affected by discouragement. Some aspects strictly related to the COVID-19 pandemic might have aggravated the mental state of old patients from our sample. These aspects are “impossibility of leaving the house,” “social isolation,” “apprehension toward the possibility of a relative getting sick,” “alarmist information on the exposure risks,” and “apprehension that oneself might be infected.” This composite picture does not appear to be a fortuitous event. Caregiver stress often has been related to emotionally charged responses characterized by exhaustion, anxiety, and irritability in the COVID-19 outbreak, and this phenomenon may induce the caregiver to misinterpret the patient symptoms, especially when behavioral disturbances are assessed by instruments configured with objective measurements (34, 35). Thus, assessment of psychopathological manifestations of a patient or informant deserves caution in this pandemic, especially through distance communication techniques, in the absence of face-to-face interaction.

According to scientific literature and reports from several countries, there is evidence that COVID-19 illness is progressively associated with underlying mental and neurological disorders, for example, anxiety, depression, sleep disturbances, delirium, dizziness, seizures, stroke, and hyposmia (39, 40). These findings bring on great concern since more than 20% of people over 60 years are affected by such preexisting illnesses (40).

As documented, after discharge patients struck by COVID-19 continued to present mental changes, including depression, anxiety, and post-traumatic stress, with high frequency and clinical relevance (41, 42). Even when the frequency does not appear to be high, the symptoms cause a significant impact on the patient's mental state. According to a large sample of 40,469 individuals with COVID-19, captured by real-time electronic records data from healthcare settings, 22.5% had any neuropsychiatric manifestations (43). In addition, they suffered from anxiety (4.6%), mood disorders (3.8%), sleep changes (3.4%), and suicidal ideation (0.2%).

Although the issue of news broadcasting by the media was not the focus of our work, it is an issue to be considered from the point of view of mental health. The excessive time spent on news exposure, the discrepancy between the amount and quality of information, conflicting messages, and some overdone coverage may induce mental changes in vulnerable populations. Li et al. (44) examined the prevalence of anxiety and depression symptoms associated with the time spent per day on news about the COVID-19 pandemic. The total prevalence was 20.4%, and rates ranged from 17.8% among individuals spending <5 min/day on news to 27.9% among those spending more than 1 h/day. Three psychosocial stressors—concern about infection; concern with income, work, and study; and disturbances caused by home quarantine—were significantly associated with the occurrence of anxiety and depression.

In unfavorable circumstances determined by the pandemic, the most fragile people may undergo sudden changes in their daily routine, with significant repercussions in psychological and behavioral status. Acute episodes of anxiety, panic attacks, irrational fears, paranoid convictions and strange behaviors, or silent resignation are common reactions converging to the fight-or-flight phenomenon (45). Elderly patients suffering from dementia or other neuropsychiatric conditions, as well as those with DS, in fact are strong candidates to display similar disorders. The mental state worsens with the increased risk related to death of family members, social isolation, difficulties in health care, and also with the profound and certainly long-term economic crisis arising. Together, these variables have been converging to an increased sense of uncertainty and helplessness among vulnerable people (45).

Caregiver burden and severity of neurocognitive diagnosis in people with DS, especially those with dementia, require better management of care during the pandemic. We know that people with dementia require different management in situations of stress (46). Despite the absence of scientific data on how the caregiver of the person with DS manifests psychological symptoms during the period of social isolation, it is known that these caregivers feel stressed and manifest psychological symptoms such as depression and anxiety (47) during the provision of care.

During the pandemic, caregivers of people with DS and dementia are likely to experience situations of increased stress due to the change in routine, requiring a change in the support model offered and better management of care provision in this situation outside the usual context (48). It is known that the effects of social isolation, in addition to the routine changes, the decrease in social life, and the withdrawal from activities carried out in institutions that provide support for people with DS and their families, can corroborate the increase in symptoms such as changes of humor reported by the caregivers who participated in this study (49).

The literature does not provide data on the adaptability of people with DS to the pandemic. However, even if the person with DS is not able to maintain a global comprehension of the impact of COVID-19 contamination on society, non-suspected cases of functional decline are still able to understand instructions and follow health recommendations (50) in a functional way in their daily lives.

Our findings are in agreement with a previous study carried out in Spain by Lara et al. (33). The authors investigated 20 individuals with MCI and 20 patients with mild AD older than 60 years who had undergone a first assessment during a month before the lockdown and were reassessed 5 weeks later. They used the NPI to detect behavioral and psychological symptoms and the EuroQol-5D scale to obtain quality-of-life characteristics, through phone interviews. The authors detected that the most affected domains, with respective scores, were apathy (5.75) and anxiety (5.30) for individuals with MCI and apathy (3.75), agitation (1.50), and aberrant motor behavior (2.00) for patients with AD. In addition, they observed a significant worsening of neuropsychiatric symptoms in both groups of MCI and AD during 5 weeks of lockdown mainly in agitation, apathy, and aberrant motor behavior. Moreover, caregivers also reported worsening with respect to their health, including mental status. As our patients were in social isolation, in general for some weeks, we compared our data with the second evaluation done by Lara and colleagues. Accordingly, caregivers investigated in our research also presented a worsening of emotional dysregulation.

Among inpatients, as expected, psychiatric symptoms must be severe, not only in those with a previous psychopathological history but also in those without chronic mental disorders. Parra and colleagues (51) conducted a single-center retrospective and observational study in Spain to describe new-onset psychotic episodes in people with COVID-19. The authors analyzed 10 patients over 18 years of age assessed by the emergency and liaison psychiatry departments at a selected hospital. Highly structured delusions of prejudice, persecutory, and referential beliefs, followed by spatial and temporal disorientation, inattention, agitation, and auditory and visual hallucinations, were the most frequent events. Pathological mechanisms, which could at least in part explain new-onset psychotic phenomena, comprise direct action of the virus into the central nervous system; also, indirect consequences of infection, for example, inflammatory reactions, metabolic disturbances, hypoxia, and prolonged immobilization, and iatrogenic outcomes from pharmacological treatments against the disease were related to the emergence of psychosis (51).

Due to the greater vulnerability associated with advanced age and medical comorbidities, elderly people, when affected, are at greater risk of serious COVID-19 outcomes. Moreover, the highly recommended social isolation, in turn, induces cognitive worsening and behavior deterioration, especially among those with dementia (52). In these circumstances, coping with worsening neuropsychiatric symptoms seems to be a valuable strategy. Resilience approaches including a structured daily routine based on cognitive stimulation, regular physical exercise, adequate diet, and sleep hygiene have been strongly recommended for maintaining mental health (53). In addition, easy access to psychiatric support for people with clinically relevant depressive or anxiety symptoms, post-traumatic stress disorder, or substance abuse should be incorporated into the comprehensive management in the COVID-19 pandemic (53).

Unsurprisingly, the COVID-19 outbreak has spread out the use of telemedicine as an assertive digital technology at the moment, and it tends to figure as an efficient long-term practice through video monitoring and other remote communication systems. Noteworthily, in the current circumstances is ongoing a new medical consultation strategy different from the traditional face-to-face interaction. Gathering technological communication resources to capture relevant data for faster decision making in clinical support emerges as a new post-pandemic challenge. Further research should be structured to assess neuropsychiatric conditions in the aging adult population, as well as to design selected and effective interventions in this situation.

Our investigation aligns with the aforementioned study pointing out that patients with cognitive decline are a particularly vulnerable population in the current scenario, who needs to be able to understand to make decisions about how to behave and what should be done. Cognitive decline, apathy, depression, and other psychopathological symptoms may interfere with the ability to understand, appreciate, or respond to most behavior safety recommendations. Furthermore, patients with AD often undergo stigmatization and resent the resource constraints due to the chronic nature of their disease and specific demands [54].

Currently, data on psychiatric disorders of patients with COVID-19 are still scarce. Several challenging issues must be highlighted. An essential proposal is to continue discussing the complex factors associated with mental suffering imposed by the persistent health crisis, to implement awareness on the risk of behavioral disturbances from existing psychiatric illnesses, and to promote resilient strategies to face this challenge (12).

### Limitations

We acknowledge that the present set of data derives from an interim analysis of a preliminary sample of a larger study, and this must be viewed as a limitation. Nonetheless, it depicts the mental health status of psychogeriatric patients in the first 3 months of the Brazilian COVID-19 pandemic. Therefore, it is not certain whether the present findings and correlations will remain in the final sample that is intended to be three to four times larger. With this precaution in mind, one can speculate that the final overall picture may turn out to be even worse, as the pandemic progresses along with many direct and indirect challenges to mental health in these elderly patients.

This cross-sectional study based on a relatively small consecutive sample, without randomization, as well as the heterogeneity of diagnoses may have restricted additional comparisons and more extensive analyses. Moreover, assessments without a direct face-to-face interaction with the patient or caregiver, implicit in the distance communication techniques, may not capture the essential complexity of the psychopathological meaning. The absence of causality between the variables was another limitation determined by the methodological design, being feasible only to perform correlation analysis. The participants had different levels of education and financial income, which possibly interfered with the quality of data acquisition (e.g., utilization of technological resources that are required to fill the questionnaires). Taken together, these factors may have affected the interpretation of the results. However, the research represents only part of a large ongoing cohort study, and therefore, these methodological weaknesses should certainly be corrected.

### Strengths

The present study was conceived as a quick response to the anticipation of clinical needs from our psychogeriatric patients during the Brazilian COVID-19 pandemic. Older adults with preexisting mental and neurocognitive disorders represent an at-risk population group, who were suddenly deprived from regular medical and rehabilitation care, given the restrictive measures that were imposed by sanitary authorities. We sought to understand their emerging symptoms and needs for care, and the present set of data enabled the implementation of proactive responses from our team using telemedicine resources.

Distinct from most studies addressing psychological/psychiatric symptoms arising in population groups exposed to the COVID-19 pandemic that were based exclusively on online questionnaires, our study included telephone interviews conducted by members of our multidisciplinary team (predominantly medical doctors), by which we were able to obtain psychometric data using validated scales (i.e., HADS and NPI-Q). In addition, given that all participants are outpatients in our psychogeriatric clinic, we could obtain reliable information from medical records in order to verify the clinical diagnoses and current prescriptions.

We also understand that the disposal of a subsample of aging adults with DS (along with a larger sample of older adults with normal karyotype) is another strength of the present study. There is a growing interest of AD researchers in DS cohorts, and most studies addressing dementia in DS do not merge these two samples. Therefore, we decided to enroll both samples in this COVID-19 study because these two patient groups are clients of our psychogeriatric service, and we understand that any insights about the effect of COVID-19 pandemic on the DS subgroup would be clinically relevant.

## Conclusion

The COVID-19 pandemic triggered prompt responses and creative initiatives from the mental healthcare professionals in many countries. In the context of the pandemic, mental health became the focus of special attention with the development of interventions to assess populations at risk. Maintaining the focus on the clinical demands of COVID-19 patients, mental health also became a priority in the context of the pandemic, with interventions aimed at vulnerable groups in the general population.

The main target of this study was the comprehensive understanding of mental and behavioral changes of older adults with neuropsychiatric conditions and DS during the COVID-19 pandemic. Dementia patients presented with worse NPI scores and higher levels of caregiver burden. Neuropsychiatric symptoms such as anxiety, irritability, depression, apathy, aberrant movement disorders, and sleep disturbances were frequent and correlated with caregiver distress.

## Data Availability Statement

The raw data supporting the conclusions of this article will be made available by the authors, without undue reservation.

## Ethics Statement

This study was reviewed and approved by the Research Ethics Committee (Comitê de Ética em Pesquisa – CEP) of HCFMUSP under approval number 4.190.468. The participants provided written informed consent to participate in this study.

## Author Contributions

CP and JL: database. MP: introduction and task management. CC and LS: down's syndrome cohort. LV: statistical analysis. FS and OF: study design, development of study questionnaire, and final review of the manuscript. The LIM-27 Psychogeriatric Team cited at Acknowledgments in this paper were also actively involved in data collection and interim analyses/discussions related to the topic. All authors were involved at manuscript preparation and data collection.

## Conflict of Interest

The authors declare that the research was conducted in the absence of any commercial or financial relationships that could be construed as a potential conflict of interest.
