# Supplementary material for: Mental Health Status of Psychogeriatric Patients During the 2019 New Coronavirus Disease (COVID-19) Pandemic and Effects on Caregiver Burden
Source: Front Psychiatry. 2020 Nov 17;11:578672. doi: 10.3389/fpsyt.2020.578672 (PMC7704440; doi:10.3389/fpsyt.2020.578672)
Supplement: Supplementary file 1 [file Data_Sheet_1.PDF]

INFORMANT'S QUESTIONNAIRE: Covid-19 pandemic and mental health of elderly patients

Section 1 of 8 – Impact of the Covid-19 pandemic on mental health of patients attending an outpatient memory clinic

The objective of this project is to assess changes in the mental health status of elderly patients and research volunteers attending an outpatient clinic during the Covid-19 pandemic.

With the help of this questionnaire, we intend to examine current mental health conditions of these people, once routine medical appointments have been canceled and postponed as advocated by sanitary authorities. We expect to obtain relevant information regarding mental and behavioural reactions towards the health crisis, as well as to investigate if there are any urgent assistance demands. We also aim to understand patients' degree of awareness concerning Covid-19 pandemic and their responses with respect to the recommended protective measures.

The information must be provided by a qualified informant who will report their impressions about the patient. Such informant may be a relative, a caregiver or their legal guardian; and must be the closest and most acquainted person, who is familiarized with the patient's daily routine and habits. When the patient him/herself demonstrates reliability in delivering the required information, the informant should take them into consideration when completing the questionnaire. In the absence of a qualified informant, the patient him/herself may provide the information.

If you agree to participate in this survey, a physician or another member of the healthcare team will proceed with some questions that can be answered by telephone or by completing a questionnaire sent by e-mail.

In case the person who this form is destined to is a healthy volunteer, where it reads 'patient', please acknowledge 'research participant'.

Informant email address: ______________________________

Section 2 of 8 – Patient Identification

Patient name: ______________________________

Patient ID: ______________________________

Do you accept in participating in this survey?

( ) Yes
( ) No

( ) Not applicable (consent shall be given by the legal guardian)

Section 3 of 8 – Informant Identification (relative, caregiver or legal guardian):

Please proceed to the next section if there is no relative, caregiver or legal guardian.
None of this information will be publicized or made available to third parties.

Informant name: ______________________________

Informant ID: ______________________________

1. In what way are you related to the patient? (Please, check as many as applicable)

( ) Family member
( ) Friend
( ) Family caregiver
( ) Professional caregiver
( ) Legal guardian
( ) Other

2. If you are a family member to the patient, what is the degree of kinship?

( ) Spouse
( ) Mother / father
( ) Son / daughter
( ) Siblings
( ) Aunt / Uncle
( ) Other
( ) Not applicable

3. Do you live in the same house as the patient?

( ) Yes
( ) No

4. For how long have you lived with the patient?

( ) Less than 6 months
( ) 6 to 12 months
( ) Between 1 and 5 years
( ) Between 5 and 10 years
( ) More than 10 years

5. Do you accept in participating in this survey?

( ) Yes
( ) No
( ) Not applicable (consent shall be given by the patient)

Section 4 of 8 – Free, Informed Consent Form (FICF)

1. Research title: The impacts of the Covid19 pandemic on the mental health of elderly patients attending an outpatient memory clinic.

Responsible researcher: ____

2. Research risk evaluation:
   (x) Minimal risk

3. Presentation:
   We invite you to participate in this scientific research.
   In case you consider participating, we will provide a copy of this free, informed consent form (FICF). This document contains descriptive information on the research. We suggest that you read it carefully and discuss with family members and/or other people that you trust in order to decide if you want to participate or not. One signed version of this form must be kept with you and one copy will be filed by the responsible researcher.

   a. Justification, objectives and procedures: This survey aims to detect possible changes in the mental health state of patients attending this memory clinic and that might have been aggravated by the Covid19 pandemic and the need for social distancing. We also intend to understand the psychological and behavioral reactions, the degree of awareness and the assistance urgencies that our patients are facing in this crisis situation.

   b. Discomforts, risks and benefits: If you agree to participate, you will also answer a questionnaire by means of a telephone interview that will be conducted by the responsible physician or another member of our healthcare team. We understand there are no risks related to this activity. On the contrary, the answers will guide us into making treatment decisions that should benefit the patients themselves. When necessary, we will offer specific and individualized orientations either through teleconsultation or scheduling a hospital appointment. The participants may have access to the final results at the end of the survey.

   c. Assistance: At any step of the study, you may have access to the healthcare professionals that are responsible for the survey.

d. Free will to refuse participating or withdrawal from the study: The choice in participating or not in this research is entirely yours. You also have the right to withdraw from the study at any time, in case you change your mind. There will be no access restrictions to the usual treatment in case you decide not to participate.

e. Guarantee of secrecy and privacy: Your data will be analyzed concomitantly to those of the other participants; no identification will be publicized under any circumstance. We ask for your authorization so that the obtained data may be used in scientific publication.

f. You will receive a copy of this FICF by e-mail if you indicate this is your wish. The provided e-mail address will be used only for the purpose of sending the FICF and the research data.

Do you wish to receive a copy of this FICF by e-mail?

( ) Yes
( ) No

Section 5 of 8 – Information about the patient

In this section, the informant must provide sociodemographic information about the patient and may consult him/her whenever necessary.

1. Date of birth:

2. Biological sex:

( ) Male
( ) Female

3. The patient identifies him/herself as:

( ) White
( ) Black / African American
( ) American Indian / Alaska Native
( ) Hispanic / Latino
( ) Native Hawaiian or Other Pacific Islander
( ) Asian
( ) I'd rather not answer

4. What is the marital status of the patient?

( ) Married
( ) Widowed
( ) Divorced or separated
( ) Single

5. Did the patient have the opportunity to study?

( ) Yes
( ) No

6. Which kind of school did the patient attend?

( ) Regular school (education beginning in childhood)
( ) Adult Education Program
( ) Special education
( ) Special classroom in regular school
( ) Regular school and special education
( ) Never attended school

7. What is the educational degree of the patient? Please check only the highest level.

	Complete	Incomplete
Basic Literacy Level	( )	( )
Elementary School	( )	( )
Middle School	( )	( )
High School	( )	( )
Adult Education Program	( )	( )
Technical Education	( )	( )
University / College	( )	( )
Post-graduate	( )	( )

8. Please inform the total time (in years) during which the patient received formal education.

Description
____
____

9. What is the patient’s current occupation?

( ) Retired
( ) Professional activity with formal employment contract
( ) Independent or liberal professional, freelancer with regular labour activity
( ) Informal worker
( ) Unemployed
( ) Never worked

10. Please inform the patient’s profession (if there is one):

Description ____

____

11. How is family income constituted? Consider patient’s income as well as the earnings and contributions from other family members to defray daily expenses. Please check as many options as applicable.

	Patient	Family members
Social security program (retirement)	( )	( )
Private pension	( )	( )
Monthly wage	( )	( )
Revenue from liberal profession	( )	( )
Financial income	( )	( )
Rental income	( )	( )
Social welfare programs	( )	( )
Government emergency aid	( )	( )
Other source of income	( )	( )

12. Including all the people that live with the patient, what is the yearly income of the family in R$?

( ) 1 to 500
( ) 501 to 1.000
( ) 1.001 to 2.000
( ) 2.001 to 3.000
( ) 3.001 to 5.000
( ) 5.001 to 10.000
( ) 10.001 to 20.000
( ) 20.001 to 50.000
( ) 50.001 to 100.000
( ) 100.001 or more
( ) I would rather not answer.

13. Does the patient feel safe regarding the financial reserves which he/she will dispose of during this crisis?

( ) Yes
( ) No
( ) I don’t know
( ) I would rather not answer

14. Does the patient have any religion?
( ) Yes

( ) No

15. If you answered ‘yes’ to the previous question, could you please inform which religion:

Description ____

16. May the patient be considered a practitioner of this religion?

( ) Yes
( ) No

17. How many people live in the same residence as the patient? (Please check 10 if more than 10 people live with the patient.)

1	2	3	4	5	6	7	8	9	10
( )	( )	( )	( )	( )	( )	( )	( )	( )	( )

18. What kind of health assistance do the patient and his/her close relatives have access to?

	Public health system	Health insurance	Private assistance
Patient	( )	( )	( )
Family members	( )	( )	( )

19. Does the patient feel safe concerning the health coverage to which him/her and his/her closest relatives have access?

( ) Yes
( ) No
( ) I don’t know

Section 6 of 8 – Occurrence of Covid19 cases in the patient’s family

This section aims to assess if the patient him/herself or one of his family members has been afflicted by the Covid-19 infection.

1. Has the patient been afflicted by infection by the new coronavirus (Covid19)?

( ) Yes
( ) No
( ) I don’t know

2. Has any of the patient’s closest relatives been aflicted by infection by the new coronavirus (Covid19)?

( ) Yes
( ) No
( ) I don’t know

3. Has any of the patient’s closest friends been afflicted by infection by the new coronavirus (Covid19)?

( ) Yes
( ) No
( ) I don’t know

4. Please inform how many people in the patient’s family have been afflicted by Covid19. (Check 10 if more than 10 people were infected.)

1	2	3	4	5	6	7	8	9	10
( )	( )	( )	( )	( )	( )	( )	( )	( )	( )

5. If there were any close relatives afflicted by Covid19, do these people live in the same residence as the patient? (Please, leave it blank if no family members were infected.)

( ) Yes
( ) No

6. What kind of health assistance do the patient and his/her close relatives have access to?

	Asymptomatic	Very mild (no treatment required)	Mild (symptomatic treatment at home)	Moderate (required hospitalization)	Severe (ICU admission)	Very severe (death)
Patient	( )	( )	( )	( )	( )	( )
Relative 1	( )	( )	( )	( )	( )	( )
Relative 2	( )	( )	( )	( )	( )	( )
Relative 3	( )	( )	( )	( )	( )	( )

7. If you wish to comment on the eventual difficulties encountered by you or your relatives due to the infection by Covid19, please use the space below:

Description
____
____

Section 7 of 8 – Perceptions of the patient regarding the crisis resulting from the pandemic and the need for social distancing.

In this section, we aim to understand the degree of awareness and insight of the patient toward the current situation and how he/she is coping with the restrictive measures.

1. What is the patient's degree of awareness concerning the Covid19 pandemic?

( ) None
( ) Minimum
( ) Reasonable
( ) Good

2. How is the patient coping with the available information on the crisis?

( ) Indifferent
( ) Alert or Attentive
( ) Worried
( ) Hypervigilant

3. Through which sources of information has the patient obtained news about the pandemic? (Please check as many as applicable.)

( ) Information provided by relatives
( ) Information provided by acquainted people (*e.g.*, family, neighbors etc.)
( ) News and announcements delivered by the community (*e.g.,* churches, associations, etc.)
( ) Information received at the workplace
( ) Radio or television
( ) Newspaper or magazines
( ) Internet
( ) Portable digital media (*e.g.,* whatsapp groups, Facebook, etc.)
( ) Other sources of information

4. Please describe the patient's reactions regarding information received about the pandemic during the last month. (Check as many as applicable.)

( ) Uninterested / Does not want to know
( ) Follows the news but does not seem to understand what is happening
( ) Acknowledges there is a problem but did not change his way of acting
( ) Accepts recommendations but often needs to be reminded
( ) Is worried and accepts the recommended measures, with no exaggeration
( ) Is very worried and distressed by the situation
( ) Is visibly disturbed by the risks of being afflicted by the infection
( ) Has completely lost his mental balance as a result of the crisis
( ) None of the above

5. Do you consider that has been aggravation or deterioration of the patient's mental disorder as a result of the pandemic? (Please consider your observations during the last month.)

( ) Yes
( ) No
( ) Maybe
( ) I do not know how to inform
( ) Not applicable (there is no mental disorder)

6. If there has been any change or aggravation of the patient's mental state, do you believe this is by reason of the Covid19 crisis?

( ) Yes, the aggravation was certainly on account of the pandemic
( ) Yes, probably the pandemic has contributed to the aggravation
( ) Maybe there is a connection, but I cannot calculate how much
( ) I do not know how to inform
( ) Not applicable (there has been no change / there is no mental disorder)

7. Which of the following features related to the pandemic might influence the patient's mental state? (Please check as many as applicable.)

( ) Alarmist information on the risks that people are exposed to
( ) Apprehension toward being infected
( ) Apprehension toward having a relative getting sick
( ) Worrying about the possibility of not receiving adequate medical assistance if infected
( ) The fact of having a relative infected with the disease
( ) The fact of having a relative that died because of the disease
( ) The need for social isolation
( ) The impossibility of receiving family and friends visiting
( ) The impossibility of leaving the house
( ) Interruption of daily activities
( ) Loss of social connections and living spaces to come together
( ) The need to perform rigid hygiene measures
( ) Social consequences as a result of the crisis
( ) Economic consequences on the country
( ) Income loss
( ) Other

8. Would you like to comment on the previous question or include any other information?

Description ____

____

9. Did you or any of your family members need to stop working or was there a remuneration decline on account of the restrictive measures due to the Covid19 pandemic? Please consider only your household income.

( ) Yes
( ) No

10. If you answered 'yes' to the previous question, did this loss in family income produce difficulties to bear with daily expenses?

( ) Yes, in a severe way
( ) Yes, necessary arrangements were made
( ) No, the household budget was not affected
( ) Not applicable (there was no income decline)

11. Please use the following space to comment on the previous question.

Description ____

____

12. What is the patient's degree of awareness regarding the requirement for social distancing?

( ) High, the patient shows enough autonomy to keep social distancing
( ) Limited, supervision is needed in order to keep social distancing measures
( ) Low, the patient needs to be often reminded on the importance of social distancing
( ) Very low, the patient does not respect social distancing
( ) The patient does not agree with the need for social distancing
( ) I do not know

13. On account of the need for social distancing, was there any qualitative change in the relationships between the patient and the people he/she lives with?

( ) There has been a prominent improvement
( ) There has been a small improvement
( ) There has been no change
( ) There has been a small deterioration
( ) There has been a pronounced deterioration

14. Does the patient receive help from a caregiver?

( ) Yes, informal caregiver (relative or friend)
( ) Yes, professional caregiver
( ) No

15. In view of the restrictive measures to urban circulation and the requirement for social distancing, has the family caregiver being receiving help from other people?

( ) Yes
( ) No
( ) I do not know how to inform
( ) Not applicable (the patient does not have / does not need a caregiver)

16. In view of the restrictive measures and the eventual consequences on the patient's mental state, was there an aggravation of the main caregiver's degree of overload?

( ) Yes
( ) No
( ) I do not know how to inform
( ) Not applicable (the patient does not have / does not need a caregiver)

17. Please use the following space to report which were the main difficulties and/or limitations in caregiving when the need for social distancing was concerned.

Description ____

____

18. Which do you consider to be the patient's level of physical activity?

( ) Physically active
( ) Fairly active
( ) Sedentary

19. Did the Covid19 crisis interfere with the patient's availability for physical activity?

( ) Yes, completely (all activities were interrupted)
( ) Yes, partially (there was a reduction in frequency and intensity of physical practice)
( ) It did not interfere (alternative ways for physical practice were implemented)
( ) There was no change since the patient did not have a previous routine of physical activity
( ) The patient is more active and has intensified his physical activity routine.

20. What is the degree of awareness of the patient about the need to implement self-protection procedures?

( ) High, the patient has enough autonomy to carry out these procedures by him/herself
( ) Limited, the patient needs supervision
( ) Low, the patient needs to be often reminded of the recommendations
( ) I do not know how to inform

( ) The patient did not change his/her hygiene habits because he/she does not believe it is necessary

21. Please check the options that reflect the adjustments to the patient's behavior in view of sanitary recommendations. In a scale from zero to five, check´0´ if the patient ignores the recommended procedures;´1´ if he/she respects them only under strong insistence from other people and still demonstrates annoyance; ´2´if he/she accepts the orientations but requires constant supervision; ´3´if he/she displays good autonomy and does not need to be reminded of the recommendations; ´4´if he/she is capable of influencing other people into following the safety procedures; ´5´if he/she reacts excessively to the recommended policies; and ´9´not applicable.

	0	1	2	3	4	5	9
Has intensified hygiene care in general	( )	( )	( )	( )	( )	( )	( )
Often washes hands and face with soap and water	( )	( )	( )	( )	( )	( )	( )
Uses alcohol-based hand sanitizer	( )	( )	( )	( )	( )	( )	( )
Performs groceries' hygiene and throws away packages	( )	( )	( )	( )	( )	( )	( )
Changes clothes more frequently (at least daily)	( )	( )	( )	( )	( )	( )	( )
Leaves the shoes in a separate place or outside the house	( )	( )	( )	( )	( )	( )	( )
Hygienizes doorknobs, doorbells, and objects of frequent contact	( )	( )	( )	( )	( )	( )	( )
Avoids leaving the house	( )	( )	( )	( )	( )	( )	( )
Leaves the house only for essential activities (groceries and pharmacy)	( )	( )	( )	( )	( )	( )	( )
Uses face masks when leaves the house	( )	( )	( )	( )	( )	( )	( )
Uses face masks when exposed to other people	( )	( )	( )	( )	( )	( )	( )
Ceased receiving visitors at home	( )	( )	( )	( )	( )	( )	( )

22. Please use the following space to comment on the patient's attitudes toward sanitary recommendations.

Description

23. Please inform on the patient's level of preoccupation regarding each of the following aspects. (Check one response in each line).

	Not worried	A little worried	Worried	Very worried	Terrified
His/her own health	( )	( )	( )	( )	( )
The health of family members	( )	( )	( )	( )	( )
Consternation toward the risk of dying	( )	( )	( )	( )	( )
Apprehension that a family member might die	( )	( )	( )	( )	( )
Fear that there will be lack of food	( )	( )	( )	( )	( )
Fear that there will be lack of medication	( )	( )	( )	( )	( )
Solitude	( )	( )	( )	( )	( )
Not being able to keep in touch with family	( )	( )	( )	( )	( )
Risk of losing job or income	( )	( )	( )	( )	( )
Not being able to bear with daily expenses	( )	( )	( )	( )	( )
Indebtedness	( )	( )	( )	( )	( )
Risk of bankruptcy	( )	( )	( )	( )	( )
Country having to face economic recession	( )	( )	( )	( )	( )
Social consequences of the crisis	( )	( )	( )	( )	( )

24. Please use the following space to comment on the patient's fears and consternations toward the pandemic.

Description ____

____

Section 8 of 8 – About the mental state of the patient during the COVID-19 pandemic.

The aim of the following questions is to estimate the occurrence of mental state and/or behavior changes during the COVID-19 crisis. Please consider any relevant changes that were noticed during the last month, as compared to the previous state.

1. Have you noticed any mental state or behavioural changes in the patient by reason of the Covid19 crisis?

( ) Yes
( ) No

( ) Maybe
( ) I do not know how to inform
( ) Not applicable (the patient did not suffer from a previous mental health condition)

2. Was there any change in sleep pattern?

( ) Insomnia
( ) Excessive somnolence
( ) Decline in sleep quality
( ) Agitated sleep
( ) There was no change in sleep pattern

3. Was there any change in appetite?

( ) Reduction in appetite
( ) Increase in appetite
( ) There was no change in appetite

4. Was there any change in eating behavior?

( ) The patient is eating less than before
( ) The patient is eating more than before
( ) There has been no change in eating behavior
( ) There has been a quality decline of food choices
( ) The patient has improved his/her eating habits
( ) I do not know

5. Has the patient displayed any weight changes during the last month?

( ) Yes, there has been weight gain
( ) Yes, there has been weight loss
( ) There has been no change in weight
( ) I do not know

6. If there has been any aggravation of a previous mental health condition, please report if you have noticed any of the following symptoms. (Check as many options as applicable.)

( ) Anxiety
( ) Panic
( ) Despair
( ) Insecurity
( ) Excessive preoccupations
( ) Pessimism
( ) Discouragement
( ) Disinterest

( ) Loss of pleasure
( ) Depression
( ) Nervousness, Uneasiness
( ) Irritability
( ) Anger
( ) Euphoria
( ) Racing thoughts
( ) Excessive shopping or unnecessary expenses
( ) Inadequate or inappropriate behavior
( ) None of the above

7. Would you like to make any remarks or comments concerning the previous question?

Description ____

____

8. May any of the following symptoms have manifested due to the current circumstances of the Covid19 crisis? (Check as many options as applicable.)

( ) Excessive distrust or suspicion
( ) Unfounded accusations directed against the people the patient lives with
( ) Persecution ideas (believing other people supposedly conceal harm intentions against him/her)
( ) Guilt ideas (tendency to feel responsible for things one did not do)
( ) Ruin ideas (tendency to perceive a certain situation as worse than it really is)
( ) Hypochondriac preoccupations (distorted or exaggerated disease-related beliefs)
( ) Belief that one is immune to the disease (thinking he/she is free from risk of contamination)
( ) Auditory hallucinations (hearing voices or nonexistent noises)
( ) Visual hallucinations (experiencing ´visions´)
( ) Other kinds of hallucinations (*e.g*., smells, body sensations)
( ) Other phenomenon that suggest a distorted perception of reality
( ) None of these symptoms

9. Would you like to make any remarks, add comments or other important information concerning the previous question?

Description ____

____

10. Did the patient present with any important behavioral change? (Please check as many as applicable.)

( ) Reduction in psychomotor activity, has been less active, more reserved, and quiet
( ) Suppression of psychomotor activity, has been completely apathetic
( ) Restlessness or motor hyperactiveness

( ) Repetitive behaviors with no clear purpose
( ) Perambulation or pacing around the house, or trying to leave the house of not detained
( ) Impulse lack of control (hasty; imprudently says or does things)
( ) Verbal aggressiveness
( ) Physical aggressiveness
( ) Inadequate sexual behavior (improper acts)
( ) Changes in eating behavior
( ) There was no significant behavior change.

11. Have you noticed any unusual behavior that, although understandable with regard to the pandemic context, can still be considered disproportionate or exaggerated?

( ) Questions and/or continuous ruminations about contamination or disease-related worries
( ) Extreme preoccupation and/or cleaning rituals involving fear of contamination
( ) Excessive accumulation of food and/or hygiene products
( ) Frequent repetitive behaviors
( ) None of these behaviors have been present

12. Would you like to make any remarks, add comments or other important information concerning changes in behavior you might have noticed during the last month?

Description ____

____

13. Was there any change in the patient's cognitive and/or functional abilities?

( ) There has been no significant change
( ) There has been some improvement when compared to before the pandemic
( ) There has been a slight deterioration when compared to previous performance
( ) There has been a pronounced deterioration
( ) I do not know how to answer

14. If there has been some change concerning the patient's cognitive and/or functional abilities, please inform which of the following you have noticed. (Check as many as applicable.)

( ) The patient is more confused / disoriented
( ) The patient is more repetitive
( ) The patient is more disorganized regarding his/her own things
( ) The patient has ceased performing chores and executing his/her usual activities
( ) The patient cannot do anything by him/herself anymore
( ) The patient presented problems related to urinary and/or intestinal control
( ) Other changes (please describe in the following space)
( ) I do not know how to inform
( ) There has been no change in the patient's cognitive and functional abilities

15. Please feel free to make any remarks, add comments or other important information concerning changes (positive or negative) in cognition and functional abilities you might have noticed during the last month.

Description ____

____

16. Has the patient been taking his/her medications according to the last medical prescription?

( ) Yes
( ) No
( ) I do not know
( ) The patient does not take medications

17. Which are the prescribed, continuous use medications?

Description ____

____

18. Does the patient use any sleep medications?

( ) Yes, following medical prescription
( ) Yes, and had to raise the dosage
( ) The patient does not use this kind of medication
( ) I do not know

19. Additionally to the prescribed medication, please inform if the patient calls upon any of the following:

( ) Self-medication
( ) Phytotherapeutic medication
( ) Supplements and/or vitamin complexes
( ) None of the above

20. Has there been any change with regard to alcohol consumption or smoking? (Please check 'not applicable' if the patient does not use those substances.)

	Increase	Reduction	No change	Not applicable
Alcohol consumption	( )	( )	( )	( )
Smoking	( )	( )	( )	( )

21. If there has been an increase in the consumption of alcohol or smoking do you consider this change a problem?

( ) Yes
( ) No
( ) I do not know
( ) Not applicable (there has not been an increase in the consumption of these substances)

22. Does the patient use any illicit substance, even if occasionally?

( ) Yes
( ) No
( ) I do not know
( ) I would rather not answer

23. If the answer to the last question was 'yes', has there been an increase in consumption pattern during the last month?

( ) Yes
( ) No
( ) I do not know
( ) Not applicable (the patient does not use these substances)

24. If you wish, please feel free to make any remarks, add comments or bring forward information on aspects you consider relevant regarding health care procedures implemented by the patient during the Covid19 crisis.

Description ____

Congratulations! You have arrived at the end of the questionnaire. Thank you for dedicating time into providing such valuable information. As a last request, we invite you to write a message that reflects how the person under your care has been feeling during the current crisis. Please ask him/her: "How do you feel and what message would you like to send to other people with respect to the pandemic?"

Description ____

CLINICIAN’S QUESTIONNAIRE: Covid-19 pandemic and mental health of elderly patients

Section 1 of 6 – Impact of the Covid-19 pandemic on mental health of patients attending an outpatient memory clinic

The objective of this questionnaire is to assess relevant clinical information about the patient. The information must be collected from the patient’s medical record, thus this part of the questionnaire should be completed by the patient’s physician or another member of the healthcare team.

The HAD and NPI-Q scales should be completed during a phone interview with the informant/caregiver responsible for completing the main questionnaire. In the absence of an informant, the patient can be interviewed.

Informant email address: ______________________________

Patient name: ______________________________

Patient ID: ______________________________

Informant name: ______________________________

Section 2 of 6 – Current demand for care

Report below the informant/caregiver/patient answer regarding the existence of any urgent demand for care in the context of the covid-19 pandemic:

Is there a need for immediate intervention regarding the patient’s mental health?

Description ______________________________

____

Section 3 of 6 – Clinical information (collected from patient’s medical record)

1. In which setting care is provided?

( ) Screening (waiting list)
( ) Outpatient clinic (private)
( ) Outpatient clinic (public)
( ) Nursery
( ) Private office

2. Date of questionnaire application:

3. Current care demand:
( ) Specialized outpatient assistance
( ) Research volunteer

4. Main psychiatric diagnosis:

( ) Major depressive disorder
( ) Bipolar disorder
( ) Anxiety disorder
( ) Obsessive-compulsive disorder
( ) Schizophrenia spectrum and other psychotic disorders
( ) Neurocognitive disorder
( ) Neurodevelopmental disorder
( ) Other

5. Secondary psychiatric diagnosis:

( ) Major depressive disorder
( ) Bipolar disorder
( ) Anxiety disorder
( ) Obsessive-compulsive disorder
( ) Schizophrenia spectrum and other psychotic disorders
( ) Neurocognitive disorder
( ) Neurodevelopmental disorder
( ) Other
( ) Not applicable (no other psychiatric diagnosis)

6. Relevant information about the psychiatric diagnosis (including specific informed diagnosis)

Description ____

____

7. Does the patient report cognitive complaints or symptoms?

Description ____

____

8. Check the option with the most adequate cognitive diagnosis (syndromic):

( ) Subjective memory complaint
( ) Mild cognitive impairment (MCI)
( ) Mild dementia
( ) Dementia
( ) Severe dementia

( ) Delirium
( ) Specific cognitive impairment, non-demential
( ) Other
( ) Not applicable (no cognitive symptoms or complaints)

9. Check the option with the possible diagnosis of the neurocognitive disorder:

( ) Prodromal Alzheimer's disease (AD) - MCI due to AD
( ) Alzheimer's disease dementia
( ) Vascular cognitive impairment
( ) Vascular dementia
( ) Mixed dementia
( ) Frontotemporal dementia (behavioural variant)
( ) Primary progressive aphasia or semantic dementia
( ) Lewy body dementia
( ) Parkinson's disease dementia
( ) Other dementia with parkinsonism
( ) Normal pressure hydrocephalus
( ) Depression with cognitive decline
( ) Bipolar disorder associated cognitive decline
( ) Schizophrenia associated cognitive decline
( ) Other neurocognitive disorder
( ) Not applicable (no neurocognitive disorder)

10. Add any information regarding the neurocognitive disorder diagnosis:

Description
___
___

11. Does the patient have clinical comorbidities? Check as many as applicable.

( ) Systemic arterial hypertension
( ) Diabetes mellitus
( ) Dyslipidemia
( ) Hypothyroidism
( ) Cancer
( ) Cardiomyopathy*
( ) Nephropathy*
( ) Digestive tract disease*
( ) Respiratory disease*
( ) Osteomuscular disease*
( ) Obesity ?
( ) Cutaneous disease*
( ) Other neurologic disease*

( ) Other clinical comorbidity*
( ) Not applicable (no comorbidities)

12. Add any information regarding the existing clinical comorbidities (mainly the asterisk-marked ones):

Description

13. Does the patient use any antidepressants? Check as many as applicable.

( ) Escitalopram/citalopram
( ) Sertraline
( ) Paroxetine
( ) Fluoxetine/fluvoxamine
( ) Venlafaxine/desvenlafaxine
( ) Duloxetine
( ) Bupropion
( ) Mirtazapine
( ) Trazodone
( ) Agomelatine
( ) Tricyclic antidepressants
( ) Other
( ) Not applicable (not using antidepressants)

14. During the LAST MONTH, was there a need for dose adjustment for any of the antidepressants listed above?

( ) Yes
( ) No
( ) Not informed
( ) Not applicable (not using antidepressants)

15. Does the patient use any antipsychotics? Check as many as applicable.

( ) Aripiprazole
( ) Clozapine
( ) Olanzapine
( ) Quetiapine
( ) Other atypical antipsychotic
( ) Risperidone/paliperidone
( ) Haloperidol
( ) Zuclopenthixol
( ) Levomepromazine/periciazine

( ) Other first generation antipsychotic
( ) Not applicable (not using antipsychotics)

16. During the LAST MONTH, was there a need for dose adjustment for any of the antipsychotics listed above?

( ) Yes
( ) No
( ) Not informed
( ) Not applicable (not using antipsychotics)

17. Does the patient use any anxiolytic drugs or sedatives? Check as many as applicable.

( ) Alprazolam
( ) Bromazepam
( ) Clonazepam
( ) Diazepam
( ) Lorazepam
( ) Other long or intermediate half-life benzodiazepine
( ) Buspirone
( ) Midazolam
( ) Nitrazepam/flunitrazepam
( ) Zolpidem
( ) Other non-benzodiazepine hypnotic drug
( ) Not applicable (not using these classes of drugs)

18. During the LAST MONTH, was there a need for dose adjustment for any of the drugs listed above?

( ) Yes
( ) No
( ) Not informed
( ) Not applicable (not using these classes of drugs)

19. Does the patient use any mood stabilizer or antiepileptic drug? Check as many as applicable.

( ) Lithium
( ) Valproic acid
( ) Carbamazepine
( ) Oxcarbazepine
( ) Lamotrigine
( ) Levetiracetam
( ) Pregabalin
( ) Topiramate

( ) Other antiepileptic drug
( ) Not applicable (not using these classes of drugs)

20. During the LAST MONTH, was there a need for dose adjustment for any of the drugs listed above?

( ) Yes
( ) No
( ) Not informed
( ) Not applicable (not using these classes of drugs)

21. Does the patient use any anti-dementia drug? Check as many as applicable.

( ) Donepezil
( ) Galantamine
( ) Rivastigmine
( ) Memantine
( ) Not applicable (not using anti-dementia drugs)

22. During the LAST MONTH, was there a need for dose adjustment for any of the anti-dementia drugs listed above?

( ) Yes
( ) No
( ) Not informed
( ) Not applicable (not using anti-dementia drugs)

23. Does the patient use any of the drugs listed below? Check as many as applicable.

( ) Modafinil
( ) Methylphenidate
( ) Lisdexamfetamine
( ) Cannabidiol
( ) Melatonin
( ) Vitamin supplements

24. During the LAST MONTH, was there a need for dose adjustment for any of the drugs listed above?

( ) Yes
( ) No
( ) Not informed
( ) Not applicable (not using these classes of drugs)

25. Add any information regarding the psychopharmacological drugs currently in use.

Description

---

26. Please, report below any clinical drugs currently in use:

Description

Section 4 of 6 – Hospital Anxiety and Depression Scale (HADS)

Adapted from Zigmond & Snaith, Acta Psychiatr Scand 1983;67,361-70;

The objective of this section is to assess and rate the presence of anxiety and depression symptoms. Check the alternative which best describes how the patient has been feeling in the PAST WEEK.

1. I feel tense or 'wound up':

( ) Most of the time
( ) A lot of the time
( ) From time to time, occasionally
( ) Not at all

2. I still enjoy the things I used to enjoy:

( ) Definitely as much
( ) Not quite so much
( ) Only a little
( ) Hardly at all

3. I get a sort of frightened feeling as if something awful is about to happen:

( ) Very definitely and quite badly
( ) Yes, but not too badly
( ) A little, but it doesn't worry me
( ) Not at all

4. I can laugh and see the funny side of things:

( ) As much as I always could
( ) Not quite so much now
( ) Definitely not so much now
( ) Not at all

5. Worrying thoughts go through my mind:

( ) A great deal of the time
( ) A lot of the time
( ) From time to time, but not too often
( ) Only occasionally

6. I feel cheerful:

Most of the time
( ) Sometimes
( ) Not often
( ) Not at all

7. I can sit at ease and feel relaxed:

( ) Definitely
( ) Usually
( ) Not Often
( ) Not at all

8. I feel as if I am slowed down:

( ) Nearly all the time
( ) Very often
( ) Sometimes
( ) Not at all

9. I get a sort of frightened feeling like 'butterflies' in the stomach:

( ) Not at all
( ) Occasionally
( ) Quite Often
( ) Very Often

10. I have lost interest in my appearance:

( ) Definitely
( ) I don't take as much care as I should
( ) I may not take quite as much care
( ) I take just as much care as ever

11. I feel restless as I have to be on the move:

( ) Very much indeed
( ) Quite a lot
( ) Not very much
( ) Not at all

12. I look forward with enjoyment to things:

( ) As much as I ever did
( ) Rather less than I used to
( ) Definitely less than I used to
( ) Hardly at all

13. I get sudden feelings of panic:

( ) Very often indeed
( ) Quite often
( ) Not very often
( ) Not at all

14. I can enjoy a good book or radio or TV program:

( ) Often
( ) Sometimes
( ) Not often
( ) Very seldom

Section 5 of 6 – The Neuropsychiatric Inventory - Questionnaire (NPI-Q)

Adapted from Cumming et al. Neurology 1994;44:2308-14;

The objective of this section is to assess the occurrence of neuropsychiatric and behavioural symptoms over the PREVIOUS MONTH. Please, inform the presence and rate the severity of symptoms described in each question and the associated impact of the symptom manifestations on the caregiver.

1. Does the patient have false beliefs, such as thinking that others are stealing from him/her or planning to harm him/her in some way?

( ) No (absence of symptom).
( ) Yes, mild (noticeable, but not a significant change)
( ) Yes, moderate (significant, but not a dramatic change)
( ) Yes, severe (very marked or prominent, a dramatic change)

1a. Does this symptom affect the caregiver? Rate the distress experienced due to that symptom.

( ) No (not distressing at all)
( ) Minimal (slightly distressing, not a problem to cope with)
( ) Mild (not very distressing, generally easy to cope with)
( ) Moderate (fairly distressing, not always easy to cope with)
( ) Severe (very distressing, difficult to cope with)
( ) Extreme or Very Severe (extremely distressing, unable to cope with)

2. Does the patient have hallucinations such as false visions or voices? Does he or she seem to hear or see things that are not present?

( ) No (absence of symptom).
( ) Yes, mild (noticeable, but not a significant change)
( ) Yes, moderate (significant, but not a dramatic change)
( ) Yes, severe (very marked or prominent, a dramatic change)

2a. Does this symptom affect the caregiver? Rate the distress experienced due to that symptom.

( ) No (not distressing at all)
( ) Minimal (slightly distressing, not a problem to cope with)
( ) Mild (not very distressing, generally easy to cope with)
( ) Moderate (fairly distressing, not always easy to cope with)
( ) Severe (very distressing, difficult to cope with)
( ) Extreme or Very Severe (extremely distressing, unable to cope with)

3. Is the patient resistive to help from others at times, or hard to handle?

( ) No (absence of symptom).
( ) Yes, mild (noticeable, but not a significant change)
( ) Yes, moderate (significant, but not a dramatic change)
( ) Yes, severe (very marked or prominent, a dramatic change)

3a. Does this symptom affect the caregiver? Rate the distress experienced due to that symptom.

( ) No (not distressing at all)
( ) Minimal (slightly distressing, not a problem to cope with)
( ) Mild (not very distressing, generally easy to cope with)
( ) Moderate (fairly distressing, not always easy to cope with)
( ) Severe (very distressing, difficult to cope with)
( ) Extreme or Very Severe (extremely distressing, unable to cope with)

4. Does the patient seem sad or say that he /she is depressed?

( ) No (absence of symptom).
( ) Yes, mild (noticeable, but not a significant change)
( ) Yes, moderate (significant, but not a dramatic change)
( ) Yes, severe (very marked or prominent, a dramatic change)

4a. Does this symptom affect the caregiver? Rate the distress experienced due to that symptom.

( ) No (not distressing at all)
( ) Minimal (slightly distressing, not a problem to cope with)
( ) Mild (not very distressing, generally easy to cope with)
( ) Moderate (fairly distressing, not always easy to cope with)
( ) Severe (very distressing, difficult to cope with)
( ) Extreme or Very Severe (extremely distressing, unable to cope with)

5. Does the patient become upset when separated from you? Doeshe/she have any other signs of nervousness such as shortness of breath, sighing, being unable to relax, or feeling excessively tense?

( ) No (absence of symptom).
( ) Yes, mild (noticeable, but not a significant change)
( ) Yes, moderate (significant, but not a dramatic change)
( ) Yes, severe (very marked or prominent, a dramatic change)

5a. Does this symptom affect the caregiver? Rate the distress experienced due to that symptom.

( ) No (not distressing at all)
( ) Minimal (slightly distressing, not a problem to cope with)
( ) Mild (not very distressing, generally easy to cope with)
( ) Moderate (fairly distressing, not always easy to cope with)
( ) Severe (very distressing, difficult to cope with)
( ) Extreme or Very Severe (extremely distressing, unable to cope with)

6. Does the patient appear to feel too good or act excessively happy?

( ) No (absence of symptom).
( ) Yes, mild (noticeable, but not a significant change)
( ) Yes, moderate (significant, but not a dramatic change)
( ) Yes, severe (very marked or prominent, a dramatic change)

6a. Does this symptom affect the caregiver? Rate the distress experienced due to that symptom.

( ) No (not distressing at all)
( ) Minimal (slightly distressing, not a problem to cope with)
( ) Mild (not very distressing, generally easy to cope with)
( ) Moderate (fairly distressing, not always easy to cope with)
( ) Severe (very distressing, difficult to cope with)
( ) Extreme or Very Severe (extremely distressing, unable to cope with)

7. Does the patient seem less interested in his/her usual activities or in the activities and plans of others?

( ) No (absence of symptom).
( ) Yes, mild (noticeable, but not a significant change)
( ) Yes, moderate (significant, but not a dramatic change)
( ) Yes, severe (very marked or prominent, a dramatic change)

7a. Does this symptom affect the caregiver? Rate the distress experienced due to that symptom.

( ) No (not distressing at all)
( ) Minimal (slightly distressing, not a problem to cope with)
( ) Mild (not very distressing, generally easy to cope with)
( ) Moderate (fairly distressing, not always easy to cope with)
( ) Severe (very distressing, difficult to cope with)
( ) Extreme or Very Severe (extremely distressing, unable to cope with)

8. Does the patient seem to act impulsively, for example, talking to strangers as if he/she knows them, or saying things that may hurt people's feelings?

( ) No (absence of symptom).
( ) Yes, mild (noticeable, but not a significant change)
( ) Yes, moderate (significant, but not a dramatic change)
( ) Yes, severe (very marked or prominent, a dramatic change)

8a. Does this symptom affect the caregiver? Rate the distress experienced due to that symptom.

( ) No (not distressing at all)
( ) Minimal (slightly distressing, not a problem to cope with)
( ) Mild (not very distressing, generally easy to cope with)
( ) Moderate (fairly distressing, not always easy to cope with)
( ) Severe (very distressing, difficult to cope with)

( ) Extreme or Very Severe (extremely distressing, unable to cope with)

9. Is the patient impatient and cranky? Does he/she have difficulty coping with delays or waiting for planned activities?

( ) No (absence of symptom).
( ) Yes, mild (noticeable, but not a significant change)
( ) Yes, moderate (significant, but not a dramatic change)
( ) Yes, severe (very marked or prominent, a dramatic change)

9a. Does this symptom affect the caregiver? Rate the distress experienced due to that symptom.

( ) No (not distressing at all)
( ) Minimal (slightly distressing, not a problem to cope with)
( ) Mild (not very distressing, generally easy to cope with)
( ) Moderate (fairly distressing, not always easy to cope with)
( ) Severe (very distressing, difficult to cope with)
( ) Extreme or Very Severe (extremely distressing, unable to cope with)

10. Does the patient engage in repetitive activities such as pacing around the house, handling buttons, wrapping string, or doing other things repeatedly?

( ) No (absence of symptom).
( ) Yes, mild (noticeable, but not a significant change)
( ) Yes, moderate (significant, but not a dramatic change)
( ) Yes, severe (very marked or prominent, a dramatic change)

10a. Does this symptom affect the caregiver? Rate the distress experienced due to that symptom.

( ) No (not distressing at all)
( ) Minimal (slightly distressing, not a problem to cope with)
( ) Mild (not very distressing, generally easy to cope with)
( ) Moderate (fairly distressing, not always easy to cope with)
( ) Severe (very distressing, difficult to cope with)
( ) Extreme or Very Severe (extremely distressing, unable to cope with)

11. Does the patient awaken you during the night, rise too early in the morning, or take excessive naps during the day?

( ) No (absence of symptom).
( ) Yes, mild (noticeable, but not a significant change)
( ) Yes, moderate (significant, but not a dramatic change)

( ) Yes, severe (very marked or prominent, a dramatic change)

11a. Does this symptom affect the caregiver? Rate the distress experienced due to that symptom.

( ) No (not distressing at all)
( ) Minimal (slightly distressing, not a problem to cope with)
( ) Mild (not very distressing, generally easy to cope with)
( ) Moderate (fairly distressing, not always easy to cope with)
( ) Severe (very distressing, difficult to cope with)
( ) Extreme or Very Severe (extremely distressing, unable to cope with)

12. Has the patient lost or gained weight, or had a change in the type of food he/she likes?

( ) No (absence of symptom).
( ) Yes, mild (noticeable, but not a significant change)
( ) Yes, moderate (significant, but not a dramatic change)
( ) Yes, severe (very marked or prominent, a dramatic change)

12a. Does this symptom affect the caregiver? Rate the distress experienced due to that symptom.

( ) No (not distressing at all)
( ) Minimal (slightly distressing, not a problem to cope with)
( ) Mild (not very distressing, generally easy to cope with)
( ) Moderate (fairly distressing, not always easy to cope with)
( ) Severe (very distressing, difficult to cope with)
( ) Extreme or Very Severe (extremely distressing, unable to cope with)

Section 6 of 6 – Conclusion

Description (optional)

____

____

Name of informant responsible for completing the questionnaire:

____

____

In the examiner's opinion, what is the degree of reliability of the informant/patient responses provided?

Patient ( ) reliable ( ) unreliable
Informant ( ) reliable ( ) unreliable
